# Extracellular matrix proteins refine microenvironments for pancreatic organogenesis from induced pluripotent stem cell differentiation

**DOI:** 10.7150/thno.104883

**Published:** 2025-01-13

**Authors:** Ming Hu, Tianzheng Liu, Hui Huang, Derek Ogi, Yinfei Tan, Kaiming Ye, Sha Jin

**Affiliations:** 1 Department of Biomedical Engineering, Thomas J. Watson College of Engineering and Applied Science, Binghamton University, State University of New York (SUNY), Binghamton, New York 13902, USA.; 2 Genomics Facility, Fox Chase Cancer Center, Philadelphia, PA, USA.; 3 Center of Biomanufacturing for Regenerative Medicine, Binghamton University, State University of New York (SUNY), Binghamton, New York 13902, USA.

**Keywords:** pancreatic extracellular matrix protein, human induced pluripotent stem cells, islet organoid development, type II collagen, cell signaling pathways

## Abstract

**Rationale:** The current understanding on manipulating signaling pathways to generate mature human islet organoids with all major hormone-secreting endocrine cell types (i.e., α, β, δ, and γ cells) from induced pluripotent stem cells (iPSCs) is insufficient. However, donor islet shortage necessitates that we produce functional islets *in vitro*. In this study, we aimed to find decellularized pancreatic extracellular matrix (dpECM) proteins that leverage signaling pathways and promote functional iPSC islet organogenesis.

**Methods:** We performed proteomic analysis to identify key islet promoting factors from porcine and rat dpECM. With this, we identified collagen type II (COL2) as a potential biomaterial cue that endorses islet development from iPSCs. Using global transcriptome profiling, gene set enrichment analysis, immunofluorescence microscopy, flow cytometry, Western blot, and glucose-stimulated hormonal secretion analysis, we examined COL2's role in regulating iPSC pancreatic lineage specification and signaling pathways, critical to islet organogenesis and morphogenesis.

**Results:** We discovered COL2 acts as a functional biomaterial that augments islet development from iPSCs, similar to collagen type V (COL5) as reported in our earlier study. COL2 substantially stimulates the formation of endocrine progenitors and subsequent islet organoids with significantly elevated expressions of pancreatic signature genes and proteins. Furthermore, it enhances islets' glucose sensitivity for hormonal secretion. A cluster of gene expressions associated with various signaling pathways, including but not limited to oxidative phosphorylation, insulin secretion, cell cycle, the canonical WNT, hypoxia, and interferon-γ response, were considerably affected by COL2 and COL5 cues.

**Conclusion:** We demonstrated dpECM's crucial role in refining stem cell differentiation microenvironments for organoid development and maturation. Our findings on biomaterial-stimulated signaling for stem cell specification, organogenesis, and maturation open up a new way to increase the differentiation efficacy of endocrine tissues that can contribute to the production of biologically functional islets.

## 1. Introduction

Diabetes mellitus is a chronic metabolic disorder with serious health complications. It is predicted to be one of the leading causes of death by 2030, according to the World Health Organization 1. While islet transplantation shows promise for curing Type 1 diabetes, this treatment is unavailable to most patients due to the scarcity of donor islets. To overcome this shortage, extensive efforts have been made to generate insulin-secreting β-cells or islet-like cell clusters from induced pluripotent stem cells (iPSCs) by mimicking *in vivo* pancreatic development processes 2-6.

To achieve pancreatic endocrine differentiation, various growth factors or signaling modulators have been utilized at different stages to direct iPSCs sequentially into definitive endoderm (DE), posterior foregut (PF), pancreatic progenitor (PP), endocrine progenitor (EP), and hormone-expressing endocrine cells (EC) 3, 7, 8. Liu *et al.* screened large amounts of chemicals and growth factors to identify the best condition for PP and β-cell differentiation 9. Ma *et al.* found that some chemicals can robustly promote the expansion of PPs 10. Nair *et al.* isolated desired cell population by fluorescence-activated cell sorting to increase the maturity of β-like cells 6. Researchers have also applied AggreWell plates to generate β-cell aggregates with a desired size 5, 6. Besides, three-dimensional scaffolding microenvironments were explored for improving the maturity of stem cell-derived islet cells 11-13. Hogrebe *et al.* highlighted the recent advances in the generation of insulin-producing β cells 14. Despite these efforts 6, 9, 15, 16, the development of mature islets that are composed of all four major hormone-secreting endocrine cells from iPSCs remains incomplete. It has been reported that insulin secretion from β-cells relies not only on the metabolic signaling associated with blood glucose level, but also on the crosstalk with other islet endocrine cells, especially α and δ cells 17. Paracrine signals from adjacent islet cells also regulate β-cell function 17. Hence, it is necessary to develop a differentiation technique to engineer whole islets from iPSCs 18. In this regard, controlling microenvironments during iPSC differentiation has shown effectiveness.

The extracellular matrix (ECM) is a microenvironment of cells in a tissue made up of a complex network of hydrated macromolecular proteins and polysaccharides 19, 20. It is known that ECM provides both physiological and biochemical cues to regulate cell fate, such as proliferation, migration, differentiation, etc. 21-25. For instance, both iPSC and mesenchymal stem cells seeded onto a decellularized liver scaffold showed higher adhesion, spatial cell distribution, albumin, and CYP450 expression 26. Therefore, mimicking a native tissue microenvironment facilitates iPSC lineage specification, differentiation, and maturation. Our previous studies demonstrated that decellularized pancreatic ECM (dpECM) and its derived molecules, such as type V collagen (COL5) and angiopoietin-2 (Ang2), promote iPSC pancreatic islet morphogenesis, organogenesis, and maturation 27, 28. These observations suggested the presence of tissue-specific cues within dpECM for pancreatic development 29, 30. In this study, to create a desired microenvironment, we investigated different types of collagens inferred by our proteomic analyses and explored tissue-specific ECM cues as functional biomaterials for enhancing islet development from iPSCs. Furthermore, we cross-examined mechanisms underlying these enhancements. This study provided new knowledge on tissue specific ECM-driven islet organogenesis.

## 2. Materials and Methods

*dpECM preparation:* Porcine pancreata were acquired from the Midwest Research Swine LLC (Gibbon, MN USA)*.* Rat pancreata were obtained from the Laboratory of Animal Resources at Binghamton University. Rats were euthanized using carbon dioxide asphyxiation before pancreata collection. All of these procedures were carried out according to the American Veterinary Medical Association guidelines. Collected pancreata were rinsed with cold PBS and stored at -80 °C until use as described in our previous study 30, 31. Procedures for decellularization, mass spectrometry for proteomic analysis were fully described in our early work 30, 31. Different batches or lot numbers of Matrigel and different individual animals with multiple preparations of decellularized pancreatic tissues were conducted for subsequent proteomics study.

*Cell culture:* Human iPSC lines IMR90 and DF4 from WiCell Research Institute were used for the study. The cells were routinely cultured in mTeSR1 medium (Stemcell Technologies) on Matrigel (MG, 80 µg/mL, Corning)-coated dishes in a 5% CO_2_ incubator at 37 °C. Cells were passaged every 2 or 3 days at ratios of 1:3 to 1:5 using dispase (Stemcell Technologies), as described in our previous study 32.

*Differentiation:* A five-stage serum-free differentiation protocol developed in our previous work was adopted as regular differentiation procedures with minor modifications by inclusion of collagen as a coating substrate 28. Different types of collagens were used as coating materials at indicated concentrations. Briefly, approximately 0.5-1 million of cells/mL were seeded on either Matrigel (MG) alone or MG blended with different types of collagens coated substrates and cultured in mTeSR1 for 24 or 48 h. The concentrations of collagen 1~5 (Sigma-Aldrich) tested were: 40 (MG + COLI~IV 40) or 80 (MG + COLI~IV 80) µg/mL. The COL5 concentrations used in this study were: 20 (MG + V20), 30 (MG + V30), and 40 (MG + V40) µg/mL. The cells were then cultured in differentiation media, as described in our previous work 28. On day 4 of Stage 4 (endocrine lineage), the cells were detached with dispase and transferred to 24-well ultra-low attachment plates for a suspension culture. Angiopoietin-2 (Ang2, Peprotech) at 20 ng/mL was supplemented to the differentiation media from Stage 4 until the end of Stage 5, as described in our early study 27. All differentiation media were exchanged every two days, except where otherwise noted. For the suspension culture, half of the medium was exchanged every other day to avoid removing aggregates. In the modified differentiation media, similar to the above five-stage differentiation procedures, 0.5% bovine serum albumin (BSA) (Sigma-Aldrich) was added to all the differentiation media. Insulin-transferrin-selenium-ethanolamine (ITS-X) (Gibco) was supplied during Stages 3~5 and 10% FBS was added at Stage 5. At Stages 3~4, the culture media contained 20 mM of total glucose and 0.5 mM sodium pyruvate. Medium compositions in regular differentiation media and modified differentiation media are shown in Table S1.

*WNT inhibition assay:* IMR90 cells were seeded on the MG, COL2 or COL5-containing MG-coated 12-well plates. The WNT inhibitor WNT-C59 (10 nM, Fisher Scientific) was added to differentiation media at Stages 2 and 3. Cells differentiated with MG-coating only and without WNT-C59 served as negative controls, while cells differentiated with COL2 or COL5 cues but without WNT-C59 acted as positive controls.

*TaqMan quantitative real-time polymerase chain reaction (qRT-PCR):* Total RNA was extracted from differentiated cells using RNeasy Mini Kit (QIAGEN) according to manufacturer's instructions. RNA was quantified by absorbance reading using Synergy H1 Microplate Reader. TaqMan qRT-PCR was performed using 100 or 200 ng RNA per reaction and QuantiTect Multiplex PCR kit (QIAGEN) on a CFX Connect Real-Time PCR system (Bio-Rad). Quality control to ensure specificity of the quantification was performed as described elsewhere in our early work 32, 33. PPIA was used as an internal control. Primer-probe details were listed in Table S2. The gene expression level was normalized to IMR90 cells by delta-delta-Ct method. At least three times of independent differentiation experiments were carried out for RNA extraction and qRT-PCR.

*Flow cytometry*: Cells were washed with PBS twice and treated with trypsin-EDTA to obtain a single-cell suspension. The cells were then fixed and permeabilized using Foxp3/Transcription Factor Fixation/Permeabilization solution (Thermo Fisher Scientific) at room temperature in the dark for 30 min. The cells were then blocked with a permeabilization buffer (Thermo Fisher Scientific) containing 5% goat serum (Gibco) at 4 °C for 30 min. For Stage 1 samples, the cells were incubated with primary antibodies or isotype antibodies (Table S3) in the permeabilization buffer at 4 °C for 1 h. For Stage 3 samples, the cells were incubated with anti-NKX6.1 antibody in the permeabilization buffer at 4 °C for 1 h, and secondary antibody at 4 °C for 1 h. The cells were then incubated with primary antibodies (Table S3) or isotype antibodies for PDX1 in the permeabilization buffer for 1 h. Finally, the cells were resuspended in PBS containing 2% bovine serum albumin (Gibco), followed by analyzing on a ZE5 Cell Analyzer (Bio-Rad). Statistics and graphing were performed using FlowJo (BD Biosciences) and gating was determined using differentiated cells stained with secondary antibodies and isotype antibodies. Details of all the antibodies used were listed in Table S3.

*Immunofluorescence microscopy:* For 2D cultured samples, cells were washed twice with PBS, fixed, and permeabilized using the Foxp3/Transcription Factor Fixation/Permeabilization solution. The cells were blocked using the permeabilization buffer containing 5% goat serum (Gibco) for 30 min, followed by staining with primary antibodies against NKX6.1, diluted in the permeabilization buffer at 4 °C overnight and then rinsed with the permeabilization buffer three times at room temperature. The samples were further stained with secondary antibodies at room temperature in the dark for 1 h. After rinsing with the permeabilization buffer three times, the cells were stained with primary antibodies against PDX1 or isotype antibodies in the permeabilization buffer for 1 h. After rinsing with the permeabilization buffer three times, the cell nuclei were counterstained with a mounting medium containing 4,6-diamidino-2-phenylindole (DAPI) (Vector Laboratories) and examined under a Zeiss 880 multiphoton laser scanning microscope. Samples processed with secondary antibodies and isotype antibodies were used as negative controls. Detailed information about the antibodies were listed in Table S4.

*Cryosectioning for immunofluorescence microscopy*: For suspension cultured samples, procedures for sample cryosectioning, antibody staining, and imaging were performed as detailed in our previous study 27. For calculating the percentage of different cell types in the organoids, images (n = 9-12) of aggregates were quantified using ImageJ software (Version 1.53t). Samples processed with secondary antibodies were used as negative controls. All the antibodies used were listed in Table S4.

*RNA-sequencing:* RNA samples were sent to LC Sciences (Houston, TX) for library preparation, mRNA-sequencing, and analysis with detailed protocols documented in our previous study 34, 35. Genes in the collagen treated group that are up- or down-regulated more than 2-fold from the control group with a *p <* 0.05 were considered significant. Heatmaps were generated at http://www.heatmapper.ca/expression/. The Kyoto Encyclopedia of Genes and Genomes (KEGG) pathway analysis was performed using DAVID Bioinformatics (https://david.ncifcrf.gov/home.jsp) 36, 37. Gene cluster plot was performed using R studio.

*Western blot analysis:* Western blot was performed as detailed in our early study 32, 34. Specifically, primary antibody was added to a buffer containing 5% nonfat milk in Tris-saline with 0.1% Tween 20, 150 mM NaCl, and 25 mM Tris-HCl (pH 7.0), and the PVDF membranes with proteins blotted were incubated at 4 °C overnight. After washing, the PVDF membranes were incubated with HRP-conjugated secondary antibody at room temperature for 1 h. A super-signal west pico plus chemiluminescent substrate (Fisher Scientific) was used to detect proteins on the membranes. Cytoplasmic and nuclear proteins were extracted using a nuclear and cytoplasmic extraction kit from Thermo Fisher Scientific. β-actin served as a loading control for the assay. The antibodies used in Western blot were listed in Table S5.

*Glucose-stimulated insulin, C-peptide, and glucagon secretion analyses*: Glucose-stimulated insulin secretion was measured as detailed in our previous study 34. Insulin stimulation index was calculated as a ratio of insulin secreted in high (20 mM) to low glucose (2 mM). Glucose-stimulated C-peptide and glucagon secretions were measured using ELISA kits from Mercodia and Millipore, respectively, according to the manufacturer's instructions and our previous study 28. Glucagon stimulation index was calculated as a ratio of glucagon secreted in low (2 mM) to high glucose (20 mM).

*Statistical analysis*: Statistical significance was calculated by unpaired two-tailed Student's t-test and *p* < 0.05 was recognized as statistically significant. Data visualization was performed using GraphPad Prism 9 (GraphPad Software Inc.). Numeric data were shown as means ± standard deviation (SD) if not otherwise specifically indicated and were derived from at least three independent experiments, and n presents the total number of independent experiments.

## 3. Results and Discussion

### 3.1. Identifying ECM that promote pancreatic progenitor development from iPSCs

**Figure 1:**
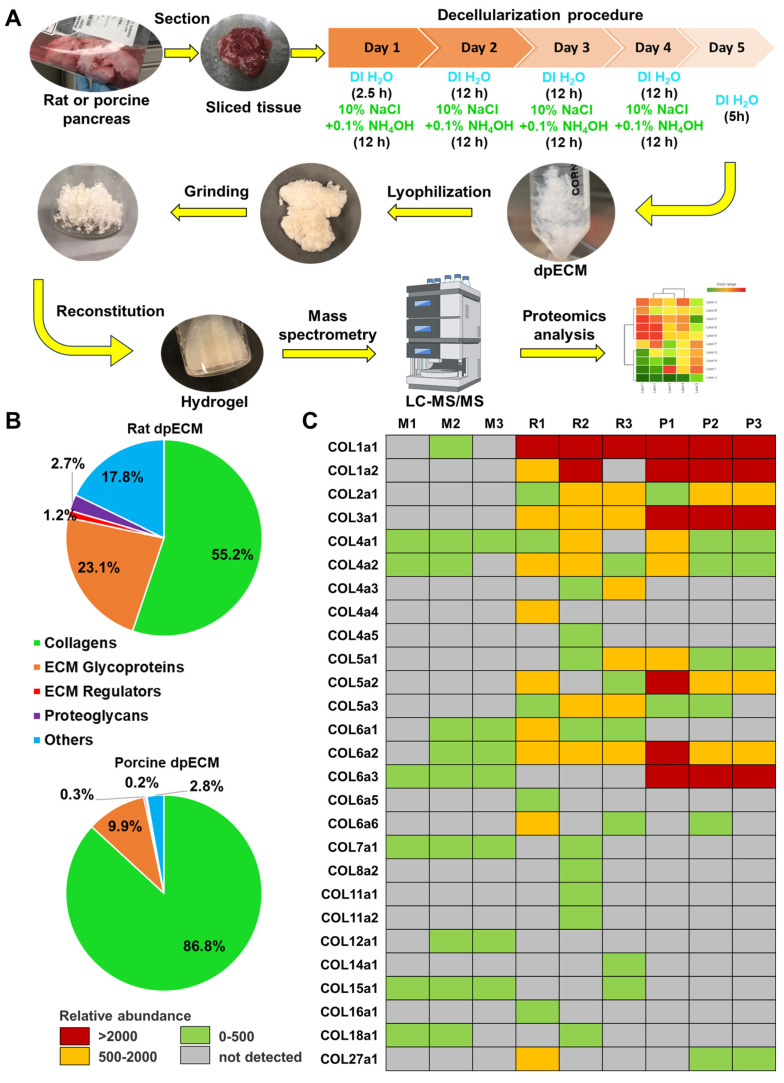
The enrichment of collagen types in rat and porcine pancreatic tissues. (A) A schematic diagram of the detergent-free decellularization procedure used for proteomics analyses (DI H_2_O: deionized water). (B) Percentage of protein abundance under different matrisome subcategories in rat and porcine dpECMs. (C) Comparison of collagen abundances among Matrigel (M1-3, n = 3) 28, rat (R1-3, n = 3) 28, and porcine (P1-P3, n = 3) dpECMs. Columns represent individual dpECM. The relative abundance of each molecule is depicted by color code.

In our previous studies, we discovered the enhancement of islet organogenesis and maturation when iPSCs were differentiated under dpECM coated substrates 28, 30. To determine which components of dpECM play a key role in this enhancement, we performed comprehensive proteomic analyses of dpECMs prepared from rat and porcine pancreata using a detergent-free decellularization approach reported in our early study (**Figure 1A**) 30, 31. We uncovered a predominance of collagen across matrisome subcategories in both species (**Figure 1B**). Among different types of collagens, collagens of type 1 ~ 5 (COL1~5) are uniquely present in dpECM but not in Matrigel (**Figure 1C**) 28, 31. Matrigel is a gelatinous ECM mixture prepared from mouse basement membrane that has been widely used in iPSC differentiation. Therefore, we selected COL1~5 for further examination. In our recent study, we have identified COL5 is one of major tissue niches presented by dpECM to boost islet development from iPSCs 28. Harnessing these successes, we intended to determine whether there are other collagen molecules playing paramount roles in promoting islet morphogenesis, organogenesis, and maturation.

**Figure 2:**
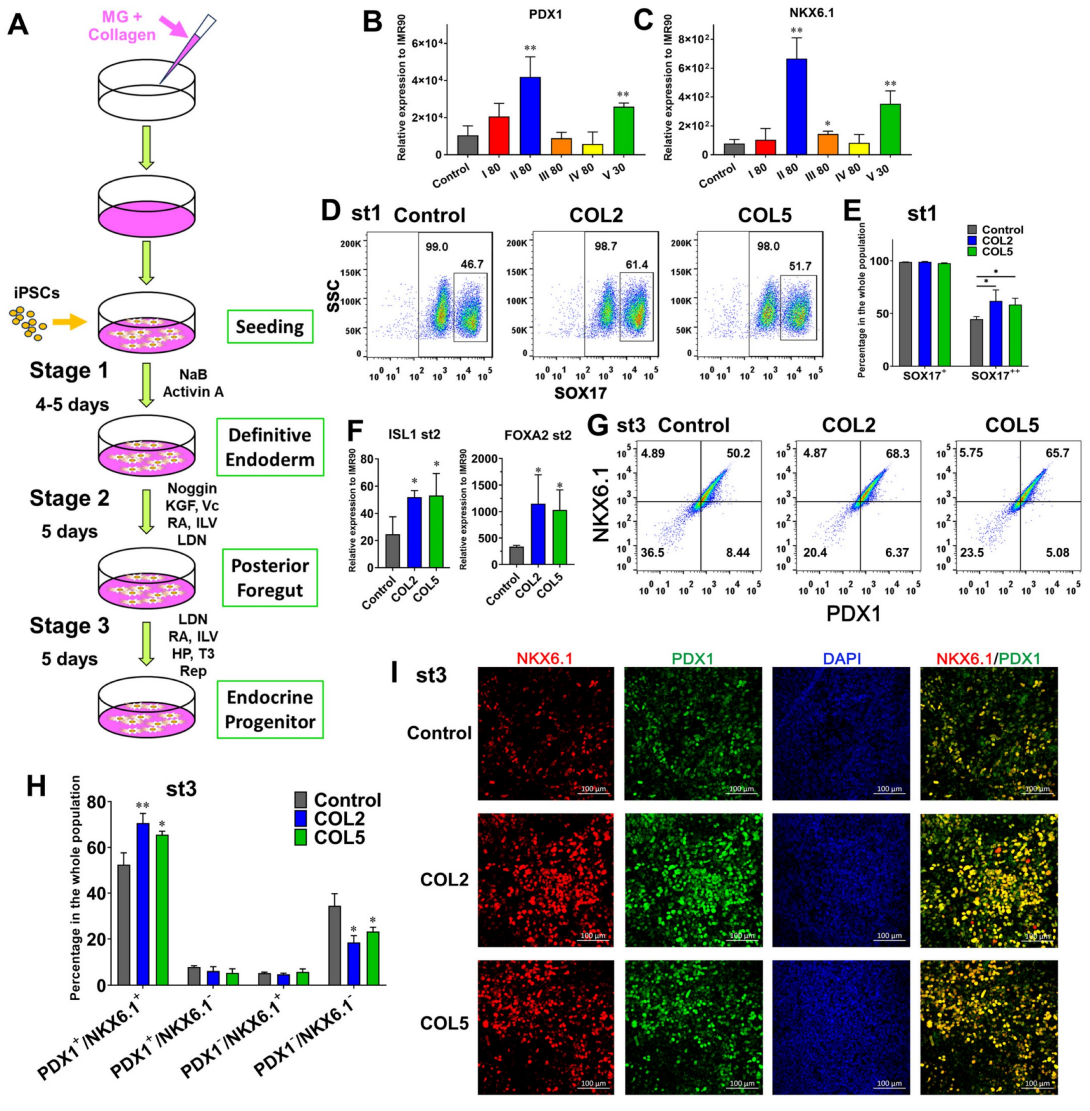
Enhancement of endocrine progenitor lineage specification from iPSCs under COL2 or COL5 stimulation. (A) A schematic diagram of a three-stage endocrine progenitor development protocol. Cells were differentiated on Matrigel (MG) as a control, or MG-collagen coated plates with indicated collagen concentration (30 and 80: 30 and 80 µg/mL). I~V denote five types of collagens. Cells were collected at the end of 3-stage differentiation, and the gene expression of PDX1 (B) and NKX6.1 (C) under different conditions were determined by real-time PCR. The expression of PDX1 and NKX6.1 were normalized to IMR90 cells (n = 3 biological replicates in each group, except n = 4 for PDX1 of control group; n = 4 for NKX6.1 of I 80 group). (D) Flow cytometric analysis of SOX17 expression on day 5. The large box shows the percentage of SOX17^+^ cells, whereas the small box indicates a higher expression level of SOX17 (SOX17^++^). (E) Comparison of percentage of SOX17^++^ cells in DE. Results are shown as mean ± SD (n = 3 biological replicates for each group). (F) The gene expression levels of ISL1 and FOXA2 on day 10 were determined and normalized to IMR90 cells (n = 4 for the control group, n = 3 for the COL2 and COL5 groups). (G) Flow cytometric analysis of PDX1^+^/NKX6.1^+^ cells collected at the end of the 3-stage differentiation. (H) Comparison of average percentage of PDX1/NKX6.1 expressing cells at the end of the differentiation (n = 3 biological replicates for each group). (B, C, E, F, H) Results are shown as mean ± SD. *, *p* < 0.05; and **, *p* < 0.01. (I) Immunofluorescence micrographs of PDX1 and NKX6.1 expressing cells at the end of the 3-stage differentiation. Cells were counterstained with DAPI (blue). Scale bar: 100 μm.

Accordingly, we tested COL1~5 sequentially to determine the effect of these collagens on pancreatic development, as they are the most enriched collagens in dpECM. We adopted a three-stage differentiation protocol and differentiated the iPSCs into pancreatic progenitors or PP on Matrigel and individual collagen type co-coated substrates. COL5 (30 µg/mL) served as a positive control. Among all COL1~5 examined, we detected a considerable elevated expression of PP markers PDX1 and NKX6.1 at the end of PP differentiation under COL2 (80 µg/mL) stimulation (**Figure 2B-C & Figure S1**). Moreover, this regulation on PP formation was dose-dependent (**Figure S1**). The PP cells generated in the COL2 group showed the highest expression level of PDX1 and NKX6.1 (**Figure 2B-C**). In contrast, COL I, III, and IV (COL1, 3, 4) had little or no effect on PP generation. To further evaluate the effect of COL2 and COL5 on PP differentiation, we quantified the protein expression of definitive endoderm or DE signature marker SOX17. While the percentages of SOX17^+^ DE cells were more than 98% in all the conditions tested (**Large box in Figure 2D & 2E**), there were approximately 62% and 59% cells expressed a high-level of SOX17 (**small box in Figure 2D & 2E SOX17^++^**) in both COL2 and COL5 groups. Only 45% SOX17^++^ cells were detected in the control group (**Figure 2E**). SOX17 expression level is a key marker for DE lineage specification during iPSC organ development. Its high-level expression suggests the DE lineage specification during iPSC organ development. The higher SOX17 expression, the more profound DE lineage is specified, thereby enhancing subsequent organ development. The two subgroups of SOX17-expressing cells shown in Figure 2D-2E indicated cells' different capacities of organ development. The COL2 or COL5 cue directed more iPSCs into SOX17 high-expressing DE cells. Furthermore, the iPSC-derived cells generated on the COL2 or COL5 substrates exhibited a 2-fold increase in the expression of PF marker ISL1 and approximately 3-fold increase in the expression of FOXA2, which is another key marker for DE and endocrine progenitors (**Figure 2F**).

In addition, we performed a flow cytometric analysis to determine the percentage of PDX1^+^ /NKX6.1^+^ cells at the end of stage 3, as these cells are able to differentiate into mature endocrine cells. As expected, cells stimulated by COL2 and COL5 demonstrated a high percentage of PDX1^+^/NKX6.1^+^. Approximately 70.6% and 65.6% of cells were PDX1^+^/NKX6.1^+^ in the COL2 and COL5 groups, whereas 52.4% of cells were PDX1^+^/NKX6.1^+^ in the control group (**Figure 2G-H**). The percentages of PDX1^-^/NKX6.1^-^ cells reduced considerably to 18.5% and 23.2% in the COL2 and COL5 groups, respectively (**Figure 2G-H**). These observations were confirmed by immunofluorescence microscopy as shown in **Figure 2I**.

To further confirm the role of COL2 and COL5 played in enhancing PP lineage specification, we induced EP differentiation using another human iPSC line DF4. We observed the similar effect of COL2 (60 µg/mL) and COL5 (30 µg/mL) on endocrine progenitor formation (**Figure S2 A**). The percentages of PDX1^+^/NKX6.1^+^ cells stimulated by COL2 or COL5 increased to 61.3% and 57.9%, respectively, as compared to those in the control group (45.7%) (**Figure S2 B-C**). The immunofluorescence microscopy confirmed the elevated expression of PDX1/NKX6.1 in the COL2 and COL5 groups (**Figure S2 D-F**).

### 3.2. Transcriptome analyses of COL2 and COL5-stimulated iPSC endocrine progenitor development

**Figure 3:**
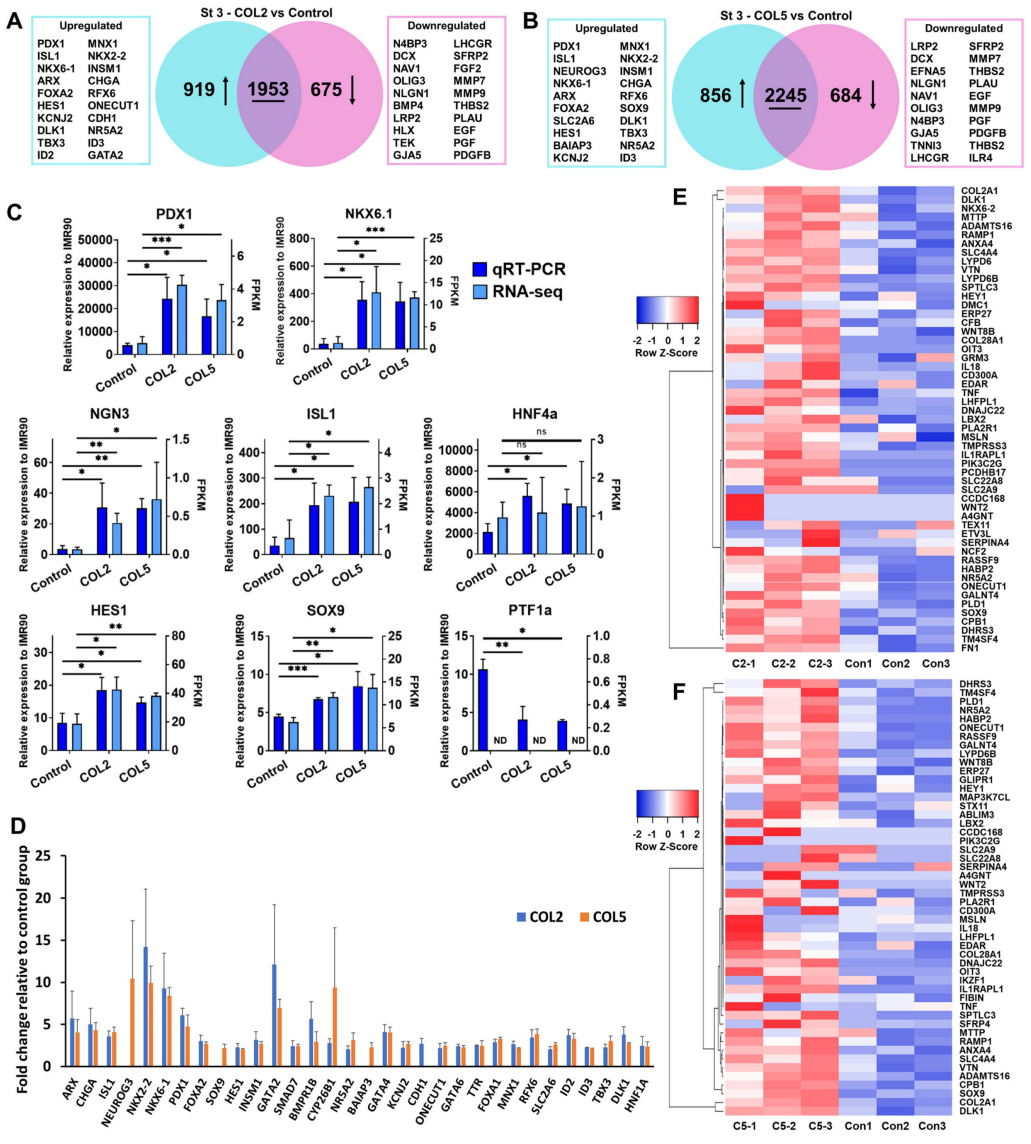
COL2 or COL5 augments endocrine progenitor development from iPSCs. iPSC IMR90 were differentiated on MG (control), or MG-COL2 or COL5 coated substrates with concentrations of 80 µg/mL COL2 or 40 µg/mL COL5. (A, B) Differentially expressed genes in the EP cells. The Venn diagrams show the number of the differentially expressed genes in the COL2 (A) and COL5 group (B). (*p <* 0.05, fold change > 2 or < 0.5) (C) qRT-PCR and RNA-seq analyses of key EP marker expression in cells collected at the end of the 3-stage differentiation. The left y-axis represents the expression levels which were normalized to IMR90 cells. The right y-axis represents fragments per kilobase of exon per million mapped (FPKM) by RNA-seq. Results are from three independent experiments and shown as mean ± SD. *, *p* < 0.05; **, *p* < 0.01; and ***, *p* < 0.001 compared to the MG group. ns: not significant; ND, not detectable. (D) RNA-seq identified upregulation of key signature genes in EP development in the COL2 or COL5 group. Results were from three independent experiments and shown as mean ± SD. (*p <* 0.05 and fold change > 2) (E-F) The heatmap of the EP signature genes that were upregulated significantly in the COL2 (E) or COL5 (F) group (*p <* 0.05 and fold change > 1.5).

To unlock underlying mechanisms of the COL2 and COL5 stimulation for directing iPSCs into EPs, we performed RNA-sequencing (RNA-seq) and mapped the differential gene expressions and their corresponding signaling pathways. We observed the upregulation of 919 and 856 genes (*p <* 0.05, fold change >2), and the downregulation of 675 and 684 genes (*p <* 0.05, fold change < 0.5) in the COL2 and COL5 groups, respectively (**Figure 3A-B**). The expressions of EP and islet markers, including NEUROG3 (also known as NGN3), PDX1, ISL1, NKX6.1, ARX, MNX1, FOXA2, SOX9, HES1, and HNF4A elevated significantly in the COL2 and COL5 groups (**Figure 3A-B**). To confirm the effect of COL2 and COL5 on the EP development, we cross-checked the expression of EP and islet signature genes by TaqMan quantitative real-time PCR (qRT-PCR) and compared the results with those obtained by RNA-seq (**Figure 3C**). The two quantitative measurements were consistent with each other in general. These genes include key EP markers, PDX1 and NGN3; pancreatic islet markers NKX6.1 and ISL1; pancreatic ductal progenitor marker SOX9; pancreatic exocrine markers HES1 and PTF1A; and β cell development and function marker HNF4A 38-41. Specifically, PDX1, NGN3, NKX6.1, ISL1, HES1, and HNF4A exhibited a 6.1, 6.0, 9.6, 5.4, 2.2, and 2.6-fold increases in the COL2 group, respectively. In the COL5 group, they were 4.7, 10.4, 9.2, 5.7, 1.7, and 2.3-fold increased, respectively (**Figure 3C**). We also observed 2.6- and 2.7-fold downregulation of PTF1A in the COL2 or COL5 group, respectively (**Figure 3C**), suggesting suppressing exocrine lineage development under these stimulations.

Moreover, we observed considerable enhanced expressions of approximately 29 and 31 genes associated with EP lineages under the COL2 or COL5 stimulation (**Figure 3D**). Most of these genes are pivotal for pancreatic endocrine development. For instance, higher expressions of NGN3, ISL1, KCNJ2, and SLC2A6 direct PP cells to differentiate toward endocrine cells 42, 43. NKX2.2 is expressed early in PP and essential for the development of islet α- and β-cells 44. Insulinoma-associated 1 (INSM1) is important in regulating the EP development. It promotes the transition from a ductal progenitor to a committed endocrine cell in developing pancreatic endocrine cells 45. Ablation of the CHGA decreases insulin cell function and enhances glucagon cell function 46. Regulatory factor X6 (RFX6) directs islet cell differentiation. Mice lacking RFX6 cannot generate normal islet cell types except for pancreatic polypeptide-producing cells 47. In addition, we found that 53 and 50 PP signature genes were significantly upregulated in the COL2 and COL5 groups, respectively (*p <* 0.05 and fold change >1.5, **Figure 3E-F**). In the cluster of significantly reduced gene expressions, these genes are associated with various non-pancreatic but other organ developmental processes according to Gene Ontology (GO) terms. For instance, genes related to nervous development, including doublecortin (DCX), neuron navigator 1 (NAV1), and oligodendrocyte transcription factor 3 (OLIG3); genes associated with kidney development, such as low density lipoprotein receptor-related protein 2 (LRP2), bone morphogenetic protein 4 (BMP4), and H2.0-like homeobox (HLX); genes in heart development, such as TEK tyrosine kinase (TEK), gap junction protein alpha 5 (GJA5), and troponin I3 (TNNI3); genes associated with male gonad development, such as luteinizing hormone/choriogonadotropin receptor (LHCGR) and secreted frizzled-related protein 2 (SFRP2) (**Figure 3A-B**) were all suppressed greatly during the iPSC pancreatic differentiation stimulated by COL2 or COL5. Overall, the iPSC-derived cells in the presence of COL2 or COL5 cues showed remarkable promotion on PP and EP development. These results clearly demonstrated the role of COL2 and COL5 played in substantially promoting the generation of EP lineage from iPSCs.

### 3.3. Unravelling signaling pathways involved in COL2 and COL5-stimulated iPSC endocrine progenitor formation

**Figure 4:**
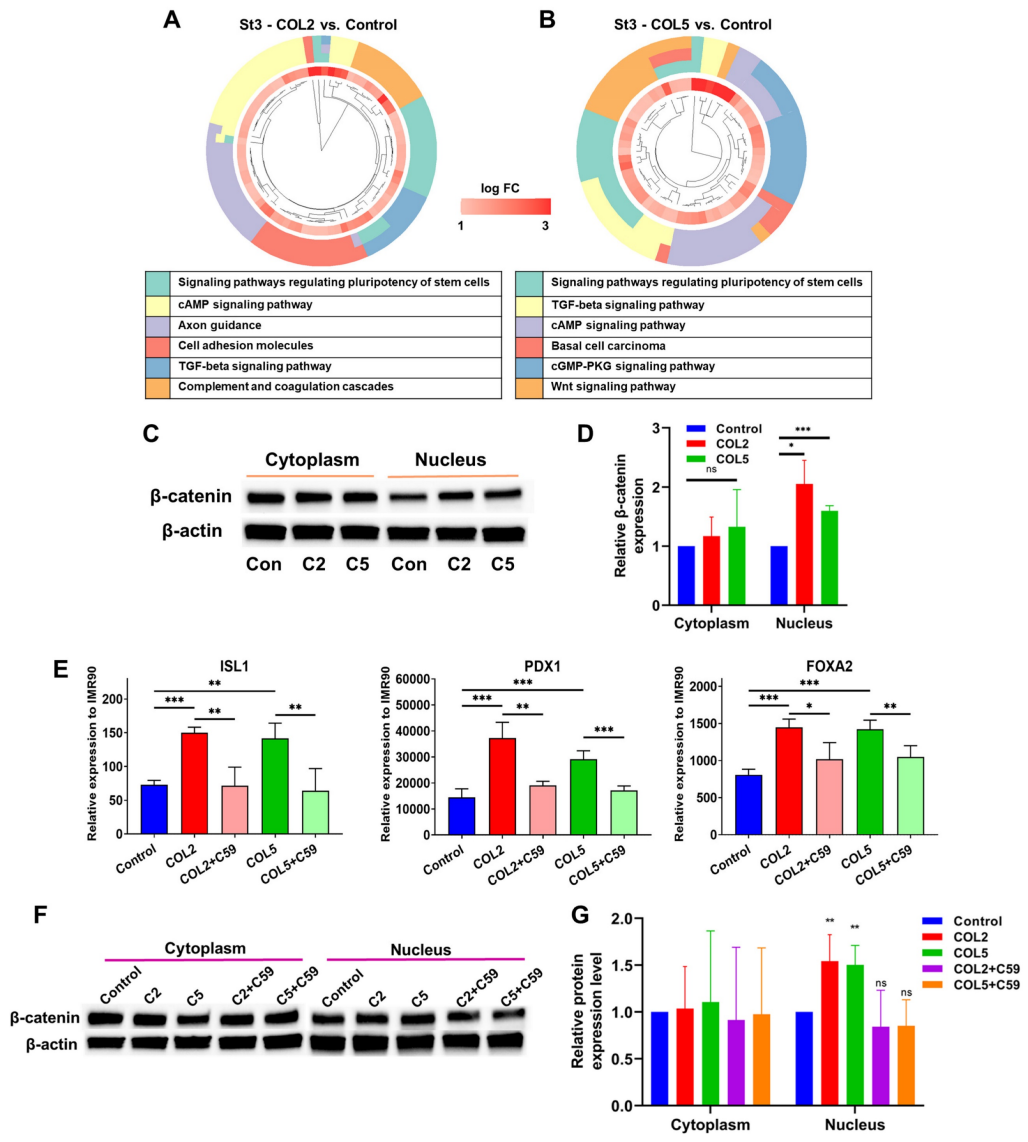
COL2 and COL5 activate the canonical WNT signaling pathway during iPSC-EP differentiation. (A-B) Gene cluster plots displaying enriched signaling pathways involved in significantly upregulated genes in cells collected under COL2 (A) or COL5 (B) stimulation. The collagen untreated iPSC differentiation served as a control. An inner circle presents the expression level of each gene shown as log fold change (log FC), whereas an outer circle depicts the signaling pathways that each gene involved in. (C) β-catenin translocation from the cytoplasm to the nucleus in the COL2 (C2), COL5 (C5), and the control (Con) groups. β-actin served as a loading control. (D) Relative β-catenin expression level after normalization to β-actin (n = 5). Results were from five independent experiments and shown as mean ± SD. *, *p* < 0.05; and ***, *p* < 0.001 compared to the control group; ns: not significant. (E) iPSCs were differentiated to EP on MG (control), MG-COL2 or COL5 coated plates. 10 nM of the WNT-C59 (C59) was supplemented to the differentiation media at Stages 2 and 3. The gene expression levels of ISL1, PDX1, and FOXA2 were detected by qRT-PCR and normalized to IMR90 cells (n = 4 biological replicates for each group). Results were shown as mean ± SD. *, *p* < 0.05; **, *p* < 0.01; and ***, *p* < 0.001. (F) The interplay between the WNT/β-catenin and COL2/COL5 cues for enhanced EP development, determined by Western blot analysis. (G) Relative β-catenin expression normalized to β-actin (n = 4). **: *p <* 0.01; ns: not significant.

Next, we conducted transcriptome analyses to unravel signaling pathways involved in the COL2 and COL5-promoted EP lineage specification. The differentially expressed genes that exhibit significant up- (fold change > 2) or down-regulation (fold change < 0.5) were subjected to GO and KEGG enrichment analyses. Notably, we discovered a number of signaling pathways that exhibited enrichment in gene clusters in both COL2 and COL5 groups. For instance, signaling pathways regulating the pluripotency of stem cells, WNT signaling, cAMP signaling, and TGF-β were enriched in both COL2 and COL5 groups (**Figure 4A-B**). Signaling pathways regulating pluripotency of stem cells play a crucial role in promoting stem cell self-renewal and pluripotency, and the downstream of these pathways include the TGF-β, PI3K-AKT, and WNT signaling pathways 48-50. The EP cells are considered self-reproducible and multipotential 51. The enrichment of these pathways under COL2 or COL5 stimulation enhances the EP cell differentiation. To validate whether the COL2 or OCL5 cue activates WNT/β-catenin signaling in the EP formation from iPSCs, we collected the cells at the end of differentiation and performed cellular fractionation analyses to detect β-catenin translocation to the nuclei by Western blotting. We observed that the expression of β-catenin within the nuclei increased in the EP cells in both COL2 and COL5 groups, which is consistent with the RNA-seq results (**Figure 4C-D**).

In contrast, the expression of cytoplasmic β-catenin was comparable across all the groups, indicating the involvement of WNT/β-catenin signaling in the enhancement of EP/PP formation under COL2 and COL5 stimulation (**Figure 4C-D**). To further validate the interplay between the COL2 and COL5 cues and WNT/β-catenin signaling for EP development, we employed a WNT inhibitor WNT-C59 (C59) to suppress the EP formation from iPSCs. C59 was added to the differentiation medium at Stages 2 and 3 to suppress the WNT signaling where COL2 or COL5 was used to stimulate islet development. C59 untreated groups, including MG-coating or COL2- or COL5-containing MG-coating groups, served as controls for comparison (**Figure 4E**). As expected, the expression of ISL1, PDX1, and FOXA2 dropped considerably in the C59 treated groups (**Figure 4E**). Furthermore, the β-catenin's nuclear shuffling was inhibited significantly by C59 as confirmed by Western blotting (**Figure 4F-G**). These results strongly suggest that the COL2 and COL5 cues promote iPSC EP differentiation by activating the WNT/β-catenin signaling pathway. The observed changes in β-catenin translocation to the nucleus coupled with the effect of WNT pathway inhibition underscored the pivotal role of the WNT/β-catenin signaling in mediating COL2 and COL5-stimulated iPSC EP development. These observations are supported by others and our early work 34, 52, 53. It is worth noting that we investigated whether treating iPSCs with both proteins during coating can further enhance the cell differentiation. However, we found no improvement in the islet development in the group of mixed COL2 and COL5 (**Figure S1 C-D**). Previous study reported by Jiang *et al.* suggested that WNT signaling activation during DE stage requires a precise control of the dose of a small molecule like CHIR99021 54. A higher dose of the small molecule reduced DE differentiation from human pluripotent stem cells. Therefore, we speculated that applying both COL2 and COL5 cues may lead to overdose in regulating WNT signaling.

**Figure 5:**
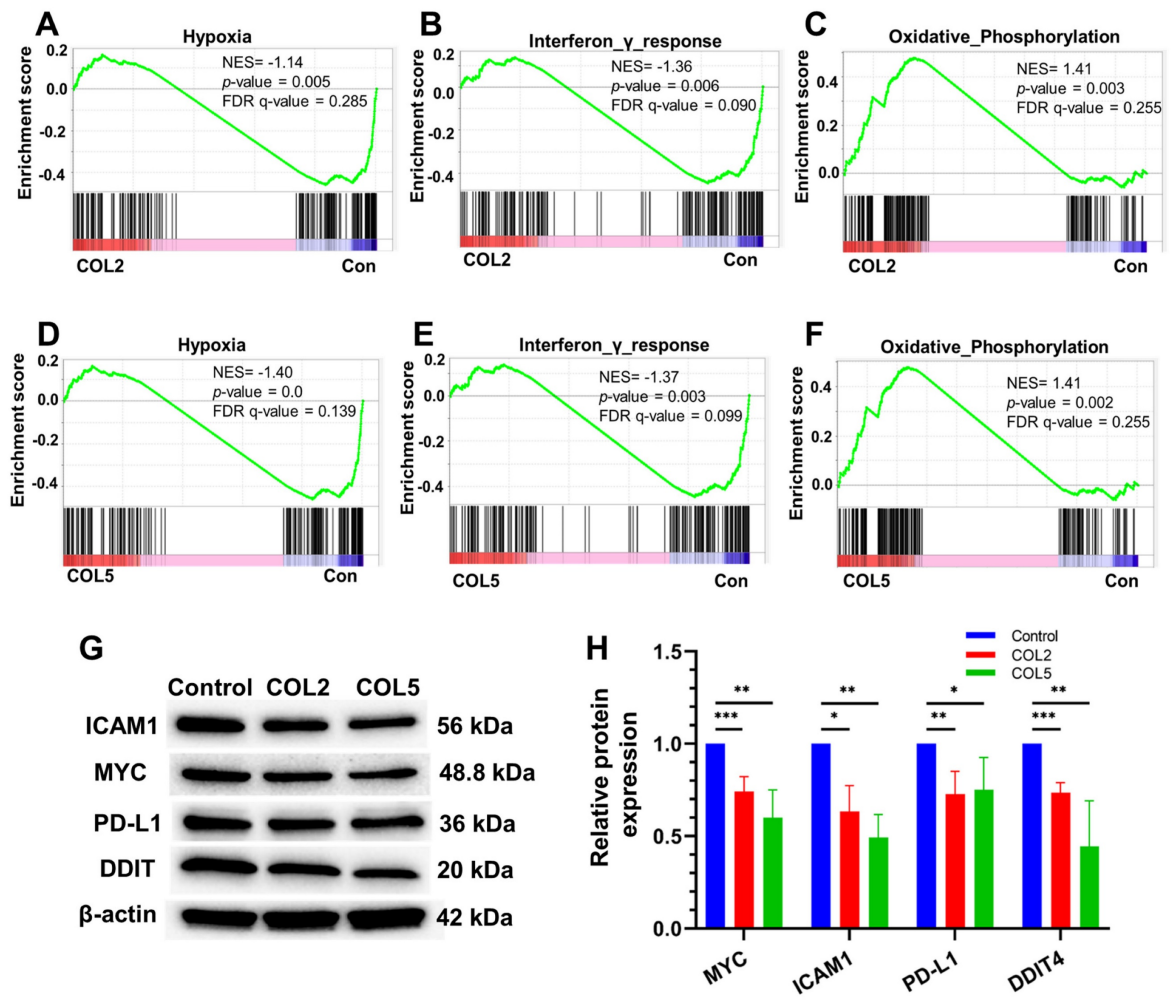
Signaling pathways involved in the COL2 and COL5 mediated regulation of iPSC-EP differentiation. GSEA showing genes associated with (A, D) hypoxia and (B, E) interferon-γ response networks were significantly enriched in EP cells in the control (MG-alone) group (Con). (C, F) Genes correlated to the oxidative phosphorylation pathway were enriched in EP cells under COL2 and COL5 cues. (G) Western blot analysis of key marker protein expression in cells harvested at the end of differentiation. (H) Relative protein expressions normalized to corresponding β-actin expression level (n = 4). *: *p <* 0.05, **: *p <* 0.01; ***: *p <* 0.001.

It is worth noting that we also carried out gene set enrichment analysis (GSEA) to explore prospective signaling pathways involved in the enhanced iPSC-EP differentiation by COL2 and COL5 niches. The GSEA results suggested that several clusters of gene expressions associated with varied signaling pathways, such as hypoxia, interferon-γ response, and oxidative phosphorylation were affected considerably by the COL2 and COL5 cues (**Figure 5A-F**). We observed a significant enrichment of genes encoding proteins that are involved in oxidative phosphorylation in the COL2 and COL5 groups (**Figure 5C & 5F**). It has been reported that iPSCs heavily rely on glycolysis for metabolism 33. As stem cells differentiate, their metabolism shifts towards oxidative phosphorylation from glycolysis 55. In addition, we found that COL2 and COL5 facilitated the iPSC-EP commitment by downregulating hypoxia-associated genes, as well as interferon-γ response network (**Figure 5A-B & 5D-E**). A hypoxic niche is beneficial to maintaining undifferentiated state of pluripotent stem cells 56, 57. The considerable suppression of hypoxia and interferon-γ response related networks in the COL2 and COL5 groups suggested that these dpECM proteins promote iPSC-EP differentiation. Furthermore, we detected protein expressions of cellular myelocytomatosis oncogene (MYC), intercellular adhesion molecule 1 (ICAM1), programmed death-ligand 1 (PD-L1), and DNA damage-inducible transcript 4 (DDIT4) by western blotting to validate these global transcriptome results (**Figure 5G-H**). MYC protein is involved in the hypoxia pathway and oxidative phosphorylation. ICAM1 and PD-L1 are related to the interferon-γ response. DDIT4 is a marker in glycolytic signaling and hypoxia pathway. The protein expression levels of MYC, ICAM1, PD-L1, and DDIT4 significantly decreased in the COL2 and COL5 groups. Overall, these observations are consistent with the results shown in **Figures 2-3**, revealing that COL2 and COL5 cues permit more efficient differentiation of iPSCs into EPs.

### 3.4. COL2 and COL5 cues promote islet organogenesis and maturation during iPSC endocrine cell differentiation

**Figure 6:**
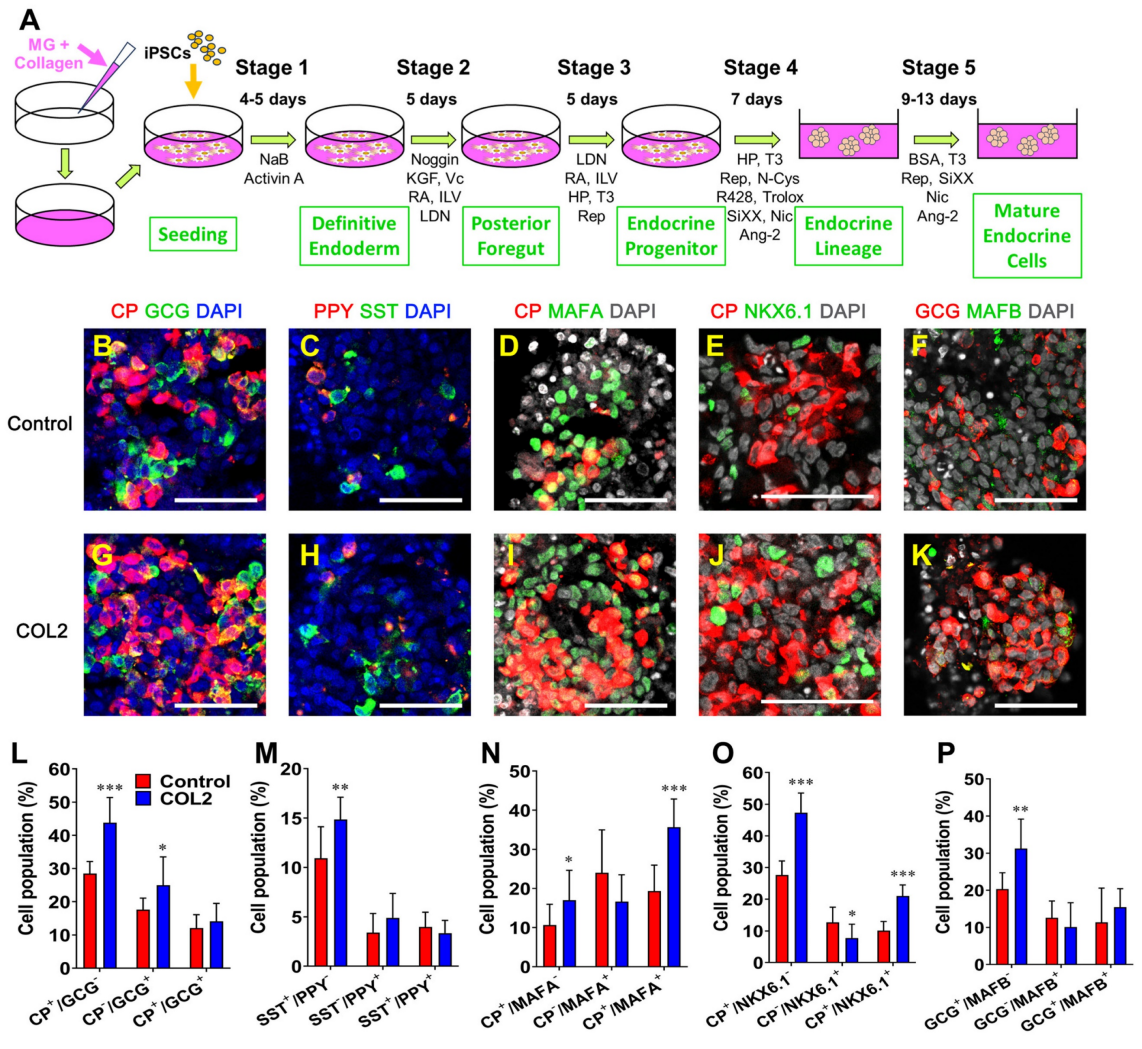
COL2 promoted the islet organogenesis and maturation from iPSCs. iPSCs were differentiated to islet organoids on MG (control), MG-COL2 or COL5 coated plates. (A) A schematic diagram of a five-stage islet development protocol. (B-K) Immunofluorescence micrographs of C-peptide (CP, red) and glucagon (GCG, green) (B, G); pancreatic polypeptide (PPY, red) and somatostatin (SST, green) (C, H); CP (red) and MAFA (green) (D, I); CP (red) and NKX6.1 (green) (E, J); and GCG (red) and MAFB (green) (F, K) in islet organoids developed under the COL 2 stimulation. The collagen untreated iPSCs served as a control. Cells were counterstained with DAPI (blue or grey). Scale bars, 50 μm. (L-P) Semi-quantitative analysis of cellularity of the islet organoids performed using ImageJ (n = 9-12 images for each condition). Results were shown as mean ± SD. *, *p* < 0.05; **p < 0.01, ***p < 0.001.

Next, we intended to examine whether the COL2 augments the assembly of pancreatic islets from iPSCs. Therefore, we adopted a serum-free, five-stage differentiation protocol from our previous study with slight modifications by exposing iPSCs to the COL2 cue. Ang2 was added to differentiation media at Stages 4~5 as shown in **Figure 6A** 27, 28.

We examined the morphology and tissue-architecture of iPSC-islets generated in the COL2 and the control group by immunofluorescence fluorescence microscopy. We observed that the localization of all four major hormone secreting islet cells, i.e., C-peptide (CP)-secreting β cells, glucagon (GCG)-secreting α cells, somatostatin (SST)-secreting δ cells, and pancreatic polypeptide (PPY)-secreting γ cells (**Figure 6B-C & 6G-H**). Mature β cell transcription factors MAFA and NKX6.1 and mature α cell transcription factor MAFB were also determined as shown in **Figure 6D-F & 6I-K**. Semi-quantification of each subtype of islet cells (n = 9-12) by ImageJ uncovered that the COL2 augmented iPSC-islet differentiation with yields of 43.8% and 25.0% CP^+^/GCG^-^ and CP^-^/GCG^+^ cells, respectively, which are significantly higher than 28.5% and 17.6% CP^+^/GCG^-^ and CP^-^/GCG^+^ cells in the control group. (**Figure 6L**). The percentage of SST^+^/PPY^-^ cells also increased considerably in the COL2 group, which was 14.9%, compared to 10.9% in the control group (**Figure 6M**). The results are similar to that obtained using COL5 reported in our previous study 28. While the population of γ cells was unaffected, the percentages of CP^+^/MAFA^+^ and CP^+^/NKX6.1^+^ populations elevated remarkably in the COL2 group, where the COL2 group generated 35.7% CP^+^/MAFA^+^ and 21.0% CP^+^/NKX6.1^+^ cells compare to 19.3% CP^+^/MAFA^+^ and 10.1% CP^+^/NKX6.1^+^ cells in the control group (**Figure 6N-P**).

**Figure 7:**
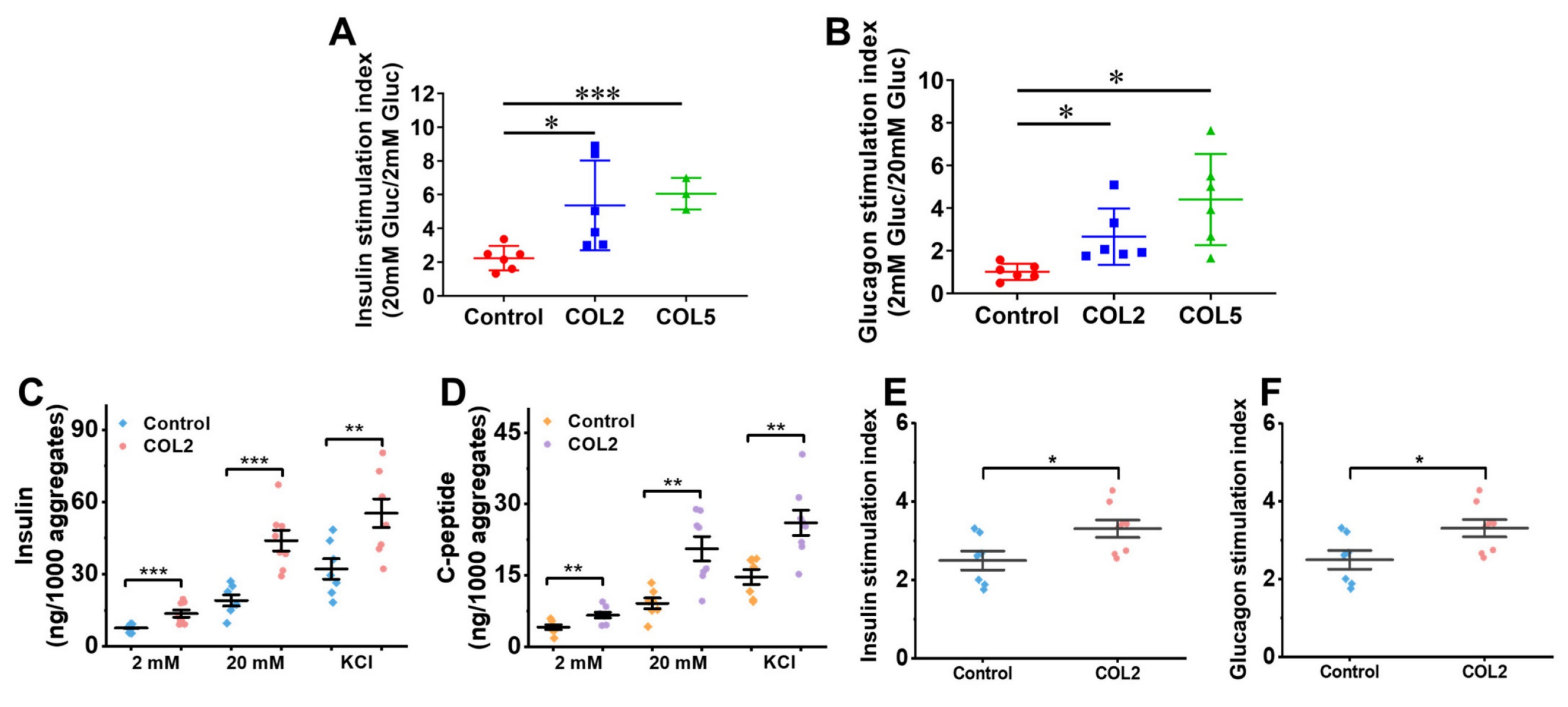
(A-B) Comparison of hormones released from the iPSC-derived islet organoids using regular differentiation media. (A) Insulin stimulation index of islets generated in the control, COL2, and COL5 groups (n = 6 for control and COL2 groups, and n = 3 for COL5 group). (B) Glucagon stimulation index of islets generated in the control, COL2, and COL5 groups (n = 6 for each group). (C-F) Comparison of hormones released from the iPSC-derived islets using modified differentiation media. The insulin (C) and C-peptide (D) released from the iPSC-derived islets (n = 8 for each group). (E-F) Insulin and glucagon stimulation indexes with modified differentiation media. Results were shown as mean ± SD. *, *p* < 0.05; **p < 0.01, ***p < 0.001.

To evaluate the biological function of the organoids generated, we performed a glucose-stimulated insulin secretion (GSIS) analysis. The results suggested that the iPSC-derived islet organoids under the COL2 or COL5 stimulation exhibited a substantially higher sensitivity to glucose changes. The insulin stimulation index, which is defined as a ratio of insulin secretion from organoids at high glucose (20 mM) to that at low glucose (2 mM), was 5.4 ± 2.7 (*p* = 0.033) and 6.1 ± 0.9 (*p* = 0.0002) in the COL2 and COL5 groups, respectively (**Figure 7A**). These results suggested that COL2 and COL5 cues promote the generation of islet organoids with more glucose-responsive insulin secretion capacity. In addition, we implemented glucose-stimulated glucagon secretion (GSGS) analysis. The glucagon stimulation index, which is defined as a ratio of glucagon secretion from organoids at low glucose (2 mM) to that at high glucose (20 mM), was remarkably increased in the COL2 and COL5 groups. It was 2.7 ± 1.3 (*p* = 0.027) and 4.4 ± 2.1 (*p* = 0.011) in the COL2 and COL5 groups, respectively (**Figure 7B**). By contrast, the glucagon stimulation index from the organoids generated in the control group was 1.0 ± 0.4, which was considered insensitive to glucose changes. Taken together, these results suggest that the COL2 and COL5 acted as functional biomaterials to stimulate mature islet organogenesis from iPSCs.

Studies from others have demonstrated a high insulin secretion capability from stem cell-derived β cells with the inclusion of serum, ITS-X and BSA in the differentiation media 5, 16. In light of the low amount of insulin secretion capability from the islet-like organoids generated under the serum-free differentiation condition, we next sought to further intensify the islet insulin secretion capacity by modifying the differentiation protocol based on recently published literatures. We have reproducibly found that the addition of the three components augments the capacity of both insulin and C-peptide release from the iPSC-islets generated under the modified differentiation conditions (**Figure 7C-D**). The islet organoids obtained under the COL2 cue together with the modified differentiation media secreted insulin at a level equivalent to approximately half of those produced by human islets at high glucose level (20 mM), as human islets secreted 80~120 ng insulin/1,000 aggregates under a high glucose level 58. Specifically, the COL2 group secreted 48.95±13.44 ng/1,000 aggregates of insulin when exposed to high glucose, which is approximately 3-fold higher than that of low glucose (2 mM) level (14.40±4.30 ng/1,000 aggregates) (**Figure 7C**). Additionally, C-peptide secretes in equimolar amounts to insulin. We also evaluated its release capacity as a secondary indicator for insulin production. The islets generated under the COL2-cue secreted 20.61±7.28 ng/1,000 aggregates of C-peptide at high (20 mM) glucose level (**Figure 7D**). Given that the molecular weight of C-peptide (3,020 Da) is approximately half that of insulin (5,808 Da), the detected C-peptide level further validated the functional insulin secretion capability of the islet organoids. These results revealed that the islets generated using the modified differentiation media along with COL2 cue facilitates the recapitulation of mature human islet organoids. In the COL2 group, the average insulin stimulation index was 3.31±0.63, which is significantly higher than the control group (2.50±0.63) (**Figure 7E**). Similarly, the C-peptide stimulation index was substantially elevated in the COL2 group (**Figure 7F**), suggesting the improvement of glucose-stimulated insulin secretion and proinsulin processing by COL2 treatment. These observations highlight that COL2 contributes to a more mature and functional endocrine phenotype in *in vitro* pancreatic islet development.

### 3.5. Systematic assessment of the effect of COL2 and COL5 on iPSC pancreatic islet development by global transcriptome profiling

**Figure 8:**
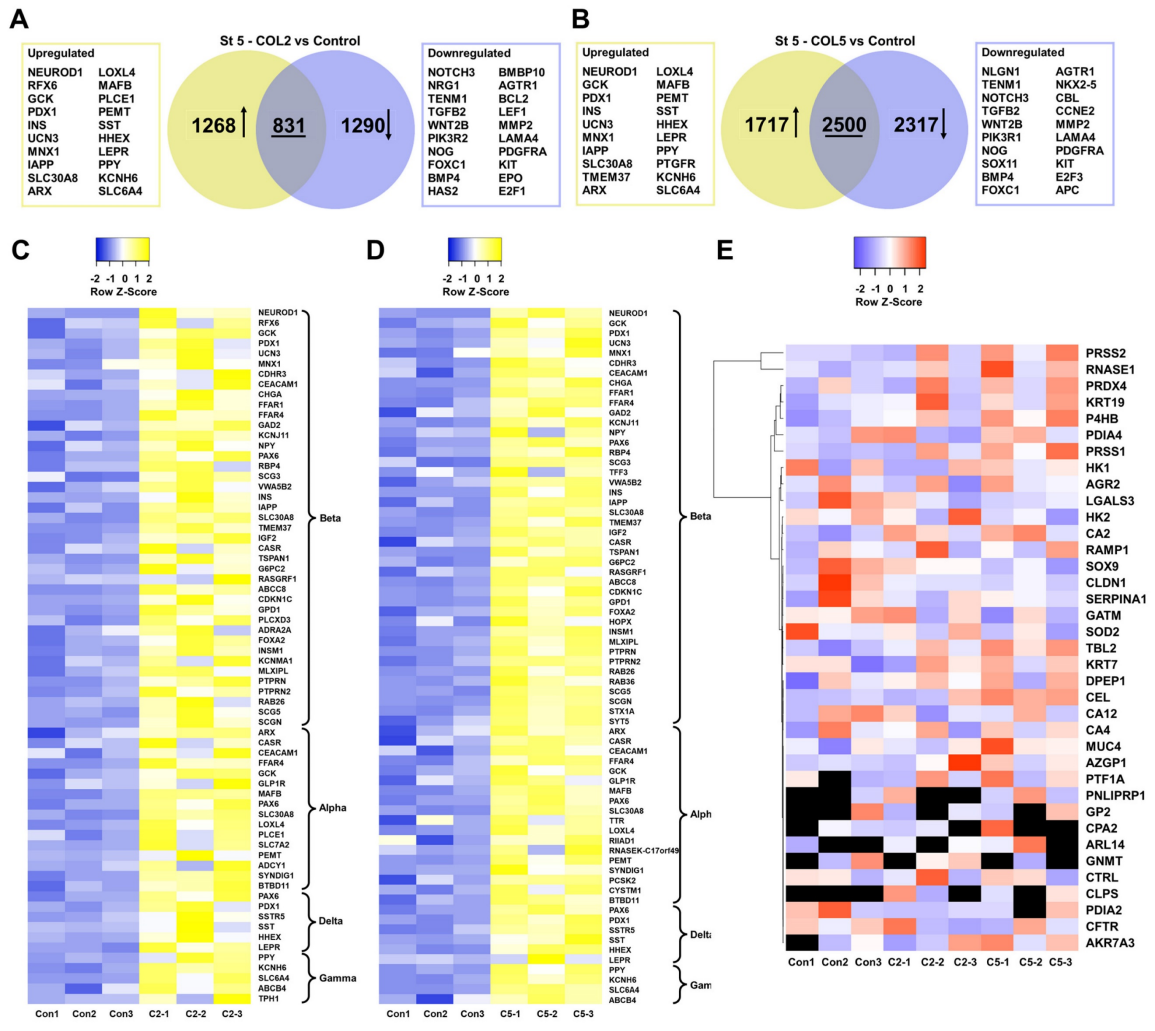
Transcriptome analysis of iPSC-islet organoid differentiation in the presence of COL2 or COL5 cue. iPSCs were differentiated to pancreatic islets on MG (control), or MG-COL2 or COL5 coated plates, with concentrations of 80 µg/mL COL2 or 40 µg/mL COL5. (A-B) Differential gene expression analysis of cells collected at end of Stage 5. Venn diagrams show the number of the significantly differentially expressed genes in the COL2 (A) and COL5 groups (B). Collagen untreated islet development served as a control. (C-D) Heatmaps of islet signature genes in the COL2 (C) and COL5 (D) groups. (E) Expression of exocrine marker genes in the control, COL2, and COL5 groups. The expression level of each gene is depicted by a color code. Undetected genes are shown in black. Results are from three independent experiments.

To systematically assess the role of COL2 and COL5 played during iPSC-islet development, we collected the cells at the end of islet development and conducted transcriptomic analyses by RNA-seq. There were 1,268 and 1,717 genes exhibiting significant upregulation (*p <* 0.05, fold change >2) and 1,290 and 2,317 genes displaying considerable downregulation (*p <* 0.05, fold change < 0.5) in the COL2 and COL5 groups, respectively (**Figure 8A-B**). Of particular interest, numerous genes upregulated in the COL2 or COL5 group were identified as islet cell markers, encompassing β cell-specific markers (NEUROD1, RFX6, GCK, PDX1, INS, etc.), α cell-specific markers (ARX, LOXL4, MAFB, PLCE1, etc.), δ cell-specific markers (SST, HHEX, and LEPR), and γ cell-specific markers (PPY, PTGFR, KCNH6, SLC6A4) (**Figure 8A-B**). Approximately 68 islet genes (41 for β, 16 for α, 6 for δ, and 5 for γ) displayed significantly elevated expressions in the COL2 group (**Figure 8C**). Similarly, around 70 islet genes (42 for β, 18 for α, 6 for δ, and 4 for γ) showed substantially enhanced expression in the COL5 group (**Figure 8D**). Interestingly, some downregulated genes were linked to various non-islet developmental processes, such as nervous (NOTCH3, NRG1, and TENM1), kidney (FOXC1, HAS2, and SOX11), or heart development (BMBP10, AGTR1, and NKX2.5) (**Figure 8A-B**), suggesting suppression of the specification of these lineages.

Additionally, we evaluated the impact of the COL2 and COL5 cues on the expression of pancreatic exocrine genes. We found that approximately 37 and 29 exocrine marker genes in the COL2 and COL5 group, respectively. These genes did not show differential gene expression as compared to the control group (*p*>0.05) (**Figure 8E**). Glycine amidinotransferase (GATM) expresses in pancreatic exocrine glandular cells with a distinct granular pattern 59. Keratin 19 (KRT19) is a cytokeratin marker specific to pancreatic duct epithelium 39. Keratin 7 (KRT7) is an exocrine duct marker gene and expresses in exocrine ducts and apico-laterally in acinar cells 60, 61. Besides, galectin-3 (LGALS3) and mucin-4 (MUC4) have been recognized as markers for pancreatic ductal adenocarcinoma 39. The transcription factor SOX9 is associated with pancreatic ductal cells 39, maintaining exclusive expression in a subset of ductal and centroacinar cells from E18.5 to adulthood during mouse pancreas development 62. Therefore, these results substantiate that COL2 and COL5 cues upregulated the expression of islet genes while leaving the expression of exocrine genes unaffected.

### 3.6. Signaling pathways involved in the COL2 and COL5-stimulated islet organogenesis from iPSCs

**Figure 9:**
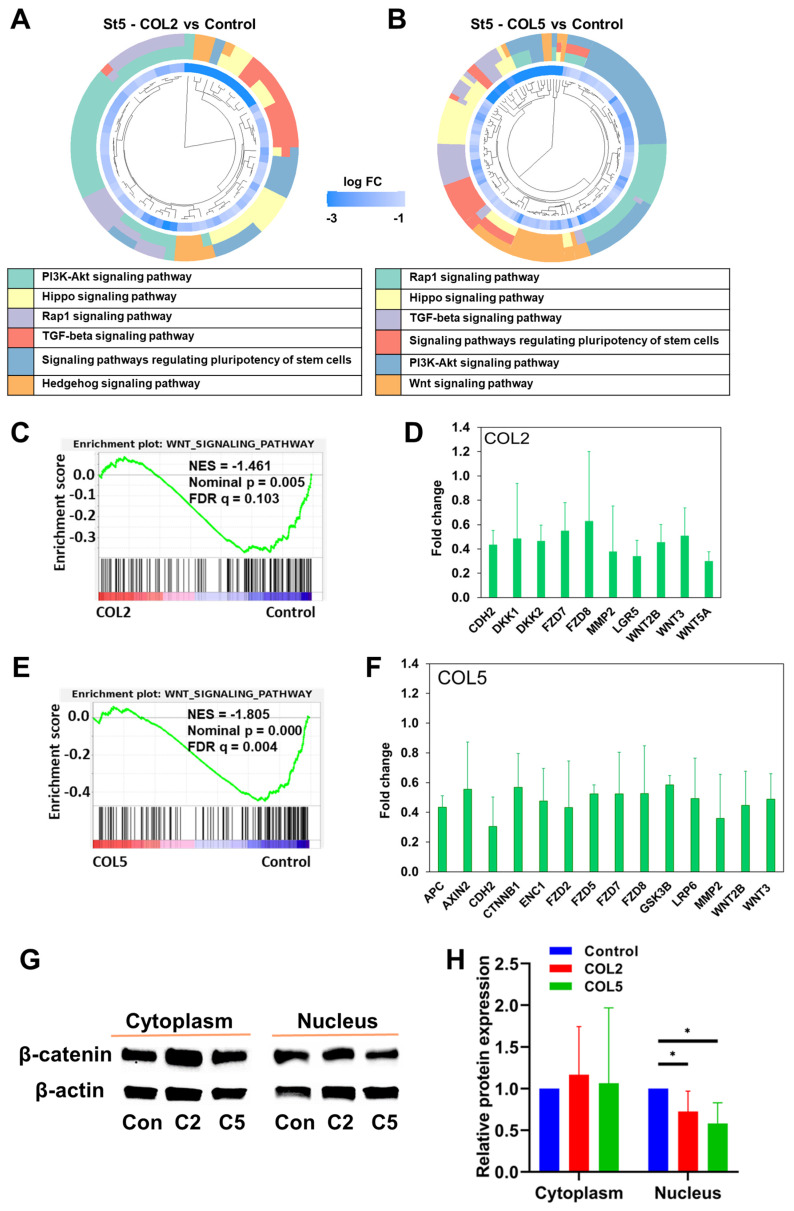
Signaling pathways involved in the COL2 and COL5-stimulated islet organogenesis. (A-B) Significantly downregulated gene clusters in the COL2 (A) and COL5 (B) groups at Stage 5 determined by KEGG analysis. The inner circle presents the expression level of each gene, shown as log FC (log fold change), and the log FC < -3 shown as the same color with log FC = -3. The outer circle presents the KEGG signaling pathways in which that each gene involved. (C-F) The downregulation of the WNT signaling pathway in the COL2 (C) and COL5 (E) groups by GSEA. (D, F) Genes involved in the canonical WNT signaling pathway were significantly downregulated (*p <* 0.05) in the COL2 (D) and COL5 (F) groups. (n = 3 in each group) (G) Western blotting of cytoplasmic and nuclear β-catenin expression in the COL2 (C2), COL5 (C5), and the control (Con) groups at Stage 5. (H) Relative β-catenin expression after normalization to β-actin (n = 5). Results were from five independent experiments and shown as mean ± SD. *, *p* < 0.05.

We performed the KEGG and GSEA to interrogate the regulatory mechanisms underlying the enhanced islet organogenesis by the COL2 and COL5 cues. We observed downregulation of signaling pathways that regulate the pluripotency of stem cells, PI3K-AKT, HIPPO, and TGF-β signaling at the end of Stage 5 (**Figure 9A-B**). These results are consistent with previous findings, as matured islet cells possess restricted proliferation capacity and the inhibition of these signaling pathways enables endocrine cell development 63-65. Previous studies demonstrated the important role of TGF-β in the generation of β-cells from human iPSC. A number of studies revealed that inhibition of TGF-β signaling could promote mature endocrine cell differentiation. Lee *et al.* highlighted this signaling in pancreatic β-cell differentiation in a comprehensive review 66. Our RNA-seq results suggested that in the stage of islet cell maturation, COL2 or COL5 treated groups showed downregulation of TGF-β signaling, which is consistent with previous finding. Besides, the WNT signaling pathway was downregulated in both the COL2 and COL5 groups (**Figure 9C-F**). Notably, the expression levels of a number of genes associated with the canonical WNT signaling pathway were decreased remarkably in both COL2 and COL5 groups. Particularly, more genes concomitant to the canonical WNT signaling pathway in the COL5 group were suppressed as compared to the COL2 group. (**Figure 9D & 9F**). To confirm the downregulation of the WNT signaling pathway at the stage of islet formation, we carried out cellular fractionation analysis to detect the involvement of WNT/β-catenin signaling by Western blotting. In accordance with the GSEA results, the expression of β-catenin protein in the cellular nuclei decreased at the end of Stage 5 in COL2 or COL5 groups (**Figure 9G-H**). Therefore, our experimental results revealed a dynamic modulation of the WNT signaling pathway from iPSCs to EP/PP and toward mature pancreatic islet cells under the COL2 and COL5 cues. These results are consistent with previous reports suggesting that inhibiting the WNT signaling pathway during β cell differentiation could enhance the yield of generated β cells *in vitro* 67, 68, while the activation of the WNT signaling facilitated iPSC differentiation into PP/EP lineages 34, 54.

**Figure 10:**
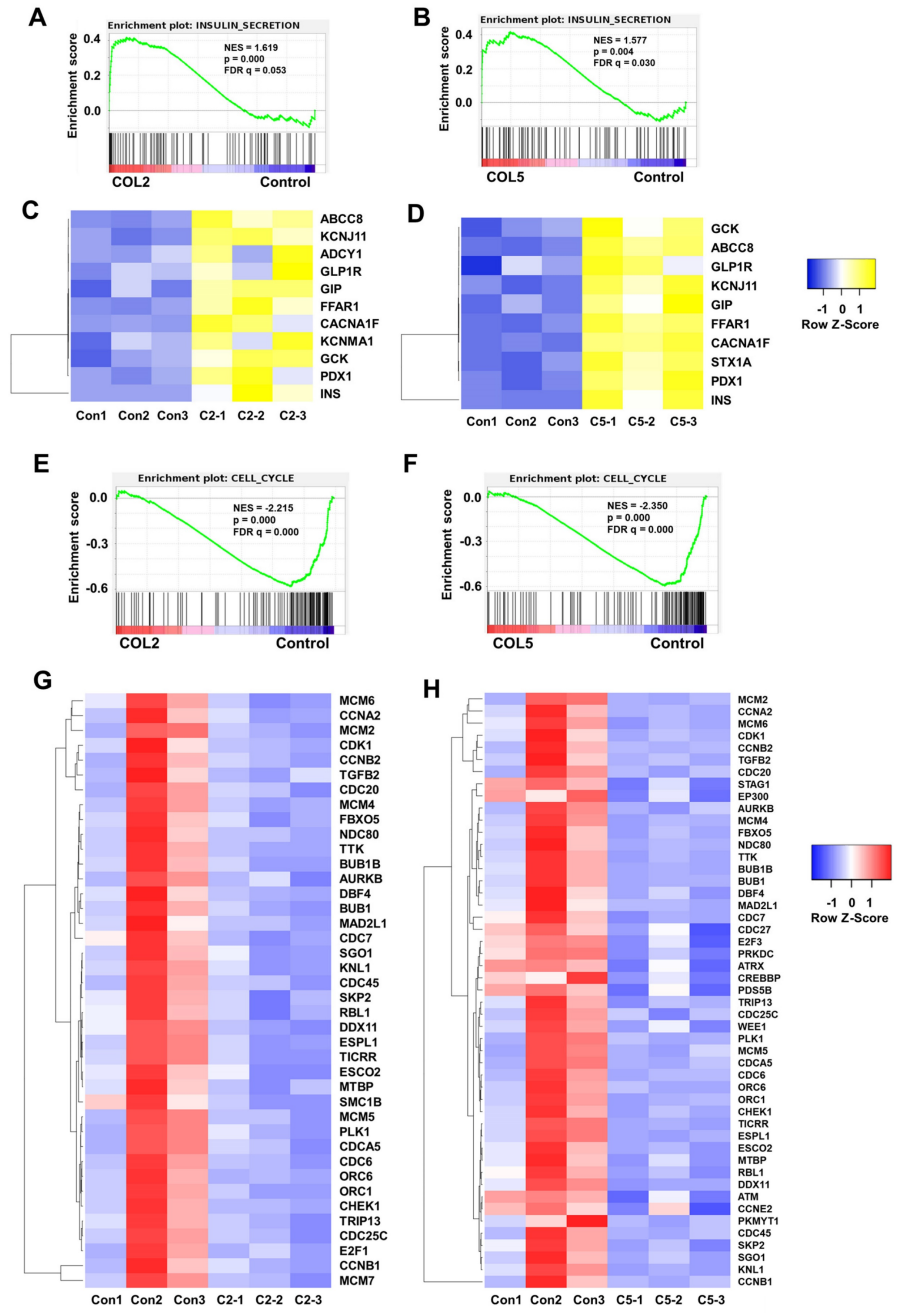
GSEA of signaling pathways involved in insulin secretion and cell cycle in the COL2 or COL5-stimulated islet organoid development. Cells harvested at Stage 5 showed the upregulation of insulin secretion in the COL2 (A) and COL5 (B) groups. Upregulation of genes involved in insulin secretion network in the COL2 (C) and COL5 (D) groups (*p <* 0.05 and fold change>2). Downregulation of cell cycle in the COL2 (E) and COL5 (F) stimulated islet development. The heatmaps of genes associated with cell cycle in the COL2 (G) and COL5 (H) groups (*p <* 0.05 and FC < 0.5). Results were from three independent experiments (n = 3 in each group).

Importantly, we observed the upregulation of insulin secretion pathway in the COL2 and COL5 groups through global transcriptome analysis (**Figure 10A-D**), which complies well with our experimental results shown in **Figure 6-8**. At least 11 and 10 genes associated with insulin secretion were upregulated significantly (fold change >2) in the COL2 and COL5 groups, respectively (**Figure 10A-D**). Notably, key β cell markers, such as INS, ABCC8, KCNJ11, FFAR1, GCK, and PDX1 were among the upregulated genes. As the insulin secretion pathway is a critical indicator of islet cell function, upregulating of this pathway demonstrated the guidance of COL2 and COL5 for iPSC-derived pancreatic islet cells maturation 69. Within this pathway, several genes, such as GIP, glucagon-like peptide-1 receptor (GLP1R) and KCNMA1, play vital roles in islet function. GIP stimulates insulin secretion of β cells in response to blood glucose level and also contributes to the survival and proliferation of β cells 70. GLP1R in pancreatic β cells promotes insulin synthesis and secretion, and β-cell survival 71, 72. Additionally, the expression of the calcium-activated large conductance subfamily M alpha member 1 (KCNMA1) indicated an enhanced insulin secretion and insulin response of β cells 73-75. In summary, the upregulation of these genes contributes to the enhanced secretion of insulin, a crucial parameter reflecting the function of mature pancreatic β cells. All these transcriptome analyses revealed mechanisms of COL2 and COL5 guiding iPSC differentiation into islets organoids.

Furthermore, GSEA revealed the downregulation of cell cycle (**Figure 10E-H**). Approximately 40 and 49 genes associated with the cell cycle pathway were significantly downregulated (fold change < 0.5) in the COL2 and COL5 groups, respectively (**Figure 10E-H**). It has been reported that inducing G1 arrest and impeding S phase are inherent characteristics of pancreatic differentiation, and exiting the cell cycle is recognized to promote the maturation of β cells 76-78. Notably, the reduction of BUB1 mitotic checkpoint serine/threonine kinase B (BUB1B) has been linked to the downregulation of mitosis and the cell cycle during β- and α-cell development 79. Inhibitors targeting minichromosome maintenance (MCM) families have shown efficacy in preventing cells from entering into the S phase and increasing the proportion of C-peptide^+^ cells in differentiated islet organoids 80. Besides, the declined or absence of the expressions of cyclin A2 (CCNA2), extra spindle pole bodies like 1 (ESPL1), aurora kinase B (AURKB), and cyclin-dependent kinase 1 (CDK1) across the entire endocrine lineage branch have been reported 77, 78, 81. Consistent with our findings, these genes in the iPSC-derived islet cells under the COL2 and COL5 cues were critically downregulated, signifying that these ECM proteins actively promote cell differentiation towards pancreatic endocrine fate.

GSEA also suggested the downregulation of signaling pathways that regulate the pluripotency of stem cells, TGF-β signaling, and DNA replication in COL2 and COL5 groups. Many genes involved in signaling pathway regulating pluripotency of stem cells displayed significant downregulation under the COL2 (C) or COL5 (D) stimulation (**Figure S3 A-D**). Shared downregulated genes in both COL2 and COL5 groups included ATP-dependent helicase 1 (SMARCAD1), frizzled class receptor 7 (FZD7), repressor element-1 silencing transcription factor (REST), FZD2, FZD8, and phosphoinositide-3-kinase regulatory subunit 3 (PIK3R3) (**Figure S3 C-D**). It has been reported that pluripotent stem cells have an elevated SMARCAD1 expression, emphasizing its role in pluripotent states 82. A high expression of FZD7 contributes to the maintenance of an undifferentiated phenotype in human embryonic stem cells 83. REST inhibition is crucial for balanced endocrine cell production from PP and induces β cell-specific genes in human adult duct-derived organoids 84. Additionally, some downregulated genes are implicated in the development of other organs. For instance, FZD2 controls limb development, FZD7 promotes skeletal muscle repair, and reduced PIK3R3 inhibits endothelial cell proliferation, migration, and angiogenesis while inducing apoptosis 85-87. Likewise, a significant decrease (fold change < 0.5) was observed in the expression of 12 and 23 genes associated with the TGF-β signaling pathway in the COL2 and COL5 groups, respectively (**Figure S4 A-D**). Studies by Xiao *et al.* demonstrated that using a TGF-β receptor I inhibitor, SB-431542 in human islets resulted in improved C-peptide secretion and an increase in β cell number through enhanced β cell proliferation, suggesting that transient suppression of TGF-β receptor signaling enhances β cell number and function 88. Moreover, Lee *et al.* revealed that inhibition of TGF-β/Smad3 signals protects β cells from apoptosis 89. Nostro *et al.* reported after induction of PP, inhibition of TGFβ/activin/nodal and BMP signaling promoted differentiation to the endocrine lineage 90.

Furthermore, a significant downregulation (fold change < 0.5) in the expression of 12 and 13 genes associated with DNA replication was observed in the COL2 and COL5 groups (**Figure S5 A-D**). The genes MCM2, MCM4, MCM5, and MCM6, are also involved in the cell cycle. MCM2-7 is a heterohexamer. It is recruited to the chromatin in the G1-phase of the cell cycle. The complex expressed in cycling cells but downregulated in quiescent cells 91. Li *et al.* proved that ESRG binds to MCM2, safeguarding error-free DNA replication and contributing to the self-renewal and pluripotency maintenance of iPSCs 92. Furthermore, DNA replication helicase/nuclease 2 (DNA2) and DNA ligase 1 (LIG1) were downregulated in both COL2 and COL5 groups. DNA2 is typically downregulated after cellular differentiation when nuclear DNA ceases replication 93. LIG1, the primary ligase in joining DNA replication intermediates, exhibits expression and activity correlating closely with the rate of cell proliferation 94. Montecucco *et al.* also reported that LIG1 mRNA decreased in cells after differentiation 95. In addition, we observed that at least 12 and 19 genes associated with oxidative phosphorylation were substantially upregulated (fold change >2) in both COL2 and COL5 groups, respectively at the end of the five-stage stepwise differentiation (**Figure S6 A-D**). This is expected, as stem cells typically possess fewer, spherical, immature mitochondria with low respiratory function, relying on anaerobic glycolysis. In contrast, differentiated cells including pancreatic cells exhibit developed mitochondrial networks with an electron-dense matrix, reduced glycolytic activity, and augmented oxidative phosphorylation activity 5, 34, 96. The enhanced oxidative phosphorylation is pivotal for glucose-stimulated insulin secretion from β cells 97. Therefore, the upregulation of these genes aligns well with our experimental results, uncovering the role of COL2 and COL5 played in promoting mature islets from iPSCs.

### 3.7. Similarities and distinctions between COL2 and COL5 acting as a functional biomaterial for iPSC-islet development

To gain an understanding of the two types of collagens as cues for pancreatic islet differentiation from iPSCs, we examined the similarity and distinction between these two biomaterials. Both promote the EP and subsequently islet development. However, we found that COL5 showed a slightly stronger regulatory effect than COL2 on EP and islet development (**Figure S7**). There were only a few genes differentially expressed significantly in the signaling pathways regulating pluripotency of stem cells, cell cycle, and oxidative phosphorylation between the COL2 and COL5 group (**Figure S7**). If setting the fold change criteria at >2 or <0.5 for comparing islets formed in the COL2 and COL5 groups, then only four gene expression levels involved in signaling pathways regulating pluripotency of stem cells were significantly different. Besides, the difference of gene expression involved in cell cycle and oxidative phosphorylation is down to only one gene. In case the fold change criteria were >1.5 or <0.67, then 5, 3, and 5 genes were found to be significantly upregulated or downregulated in each respective pathway (**Figure S7**). These outcomes aligned well with observed higher glucose sensitivity of the islets generated in the COL5 group (**Figure 7A-B**) and stronger signaling pathway modulations (**Figure 5 & 9**). Taken together, our study demonstrated that COL2 or COL5 cue regulates key marker genes and signaling pathways that are beneficial to the generation of biologically functional pancreatic islet organoids from iPSCs.

## 4. Conclusions

Our study elucidates the pivotal role of COL2 and COL5 as crucial microenvironmental cues for fostering the generation of pancreatic islets from iPSCs. The COL2 and COL5 stimulation on iPSCs resulted in the enhanced generation of PDX1^+^/NKX6.1^+^ EP cells. Immunofluorescence microscopic analysis demonstrated the promotion of insulin-secreting β cells, glucagon-secreting α cells, and somatostatin-secreting δ cells in the islet organoids. Likewise, COL2 and COL5 cues augmented organoids' function by improved insulin and glucagon secretion in response to glucose challenges. Furthermore, our transcriptomic analyses unveiled signaling pathways involved in enhanced EP and islet lineage specification under the COL2 and COL5 cues. Collectively, our findings revealed that COL2 and COL5, as functional biomaterials, are capable of driving iPSC differentiation towards EP and subsequent islet organoids, advancing stem cell research for tissue engineering and regenerative medicine. Given that other collagen types such as COL1, 3 and 4 have little or no effect on enhancing iPSC-PP differentiation, this could be future work to fully investigate the mechanisms of each collagen type on iPSC pancreatic differentiation.

## Supplementary Material

Supplementary figures and tables.
