# Supplementary material for: Extracellular matrix proteins refine microenvironments for pancreatic organogenesis from induced pluripotent stem cell differentiation
Source: Theranostics. 2025 Jan 13;15(6):2229–49. doi: 10.7150/thno.104883 (PMC11840725; doi:10.7150/thno.104883)
Supplement: Supplementary file 1 — Supplementary figures and tables. [file thnov15p2229s1.pdf]

Supplementary information

Extracellular matrix proteins refine microenvironments for pancreatic organogenesis from induced pluripotent stem cell differentiation

Ming Hu[1], Tianzheng Liu[1], Hui Huang[1], Derek Ogi[1], Yinfei Tan[2], Kaiming Ye[1,3], and Sha Jin[1,3]*

Corresponding author email: sjin@binghamton.edu

[1]Department of Biomedical Engineering, Thomas J. Watson College of Engineering and Applied Science, State University of New York (SUNY) at Binghamton, New York 13902

[2]Genomics Facility, Fox Chase Cancer Center, Philadelphia, PA, USA

[3]Center of Biomanufacturing for Regenerative Medicine, State University of New York (SUNY) at Binghamton, New York 13902

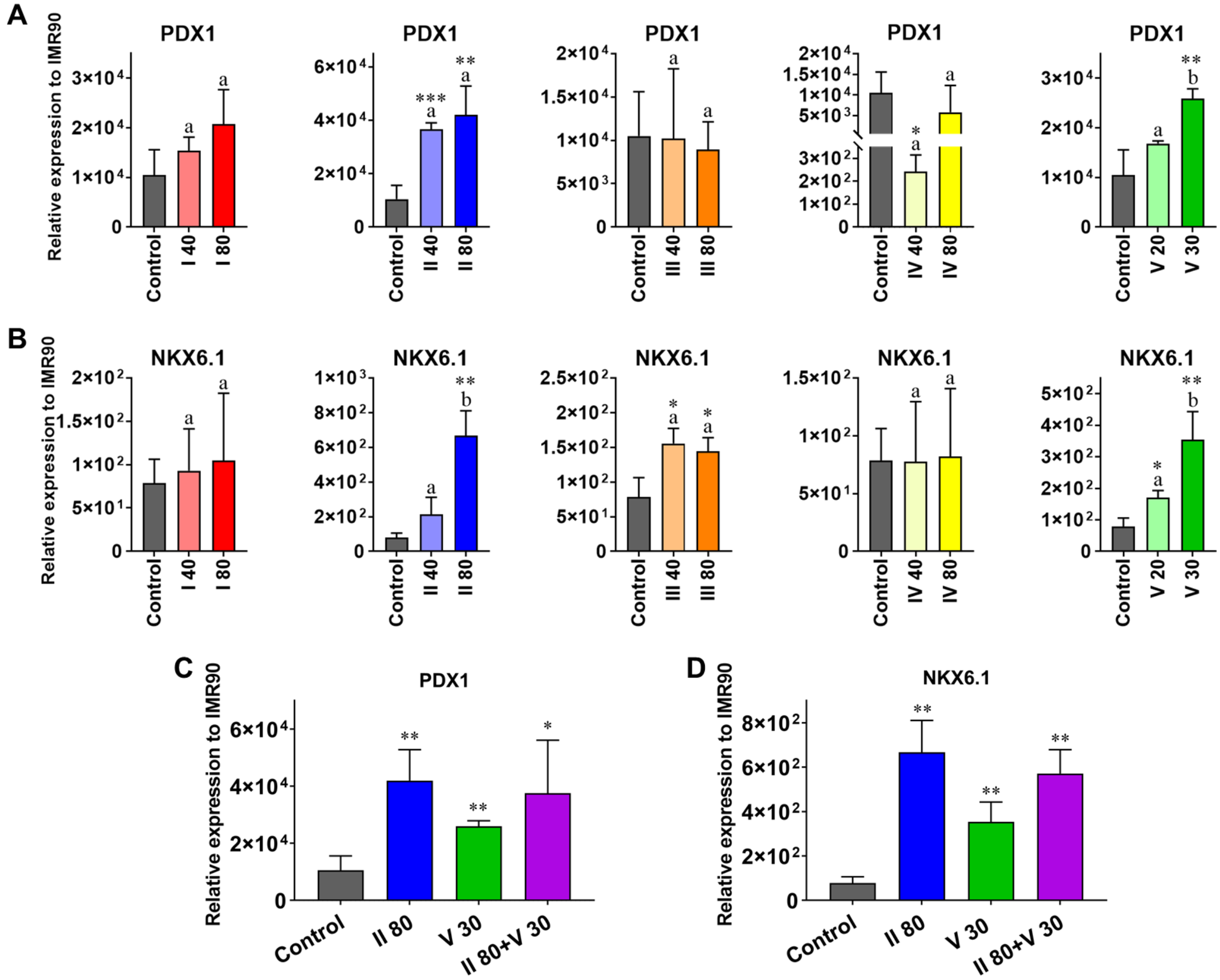

Figure S1. The effect of collagen on pancreatic progenitor lineage specification. Human iPSCs IMR90 were differentiated to endocrine progenitors on Matrigel (Control); or 20, 30, 40, or 80 µg/mL collagen I~V blended Matrigel substrates. Key marker gene expression of pancreatic progenitor PDX1 (A, C) and NKX6.1 (B, D) under various collagen stimulation after normalizing to IMR90 cells (n = 3 biological replicates in each group, except n = 4 for PDX1 in the control group; n = 4 for NKX6.1 in the I 40 and I 80 groups). Results were from three or four independent experiments and shown as mean ± SD. *, $p < 0.05$; **, $p < 0.01$; and ***, $p < 0.001$. Different letters above the bars represent that the bars are significantly different from each other.

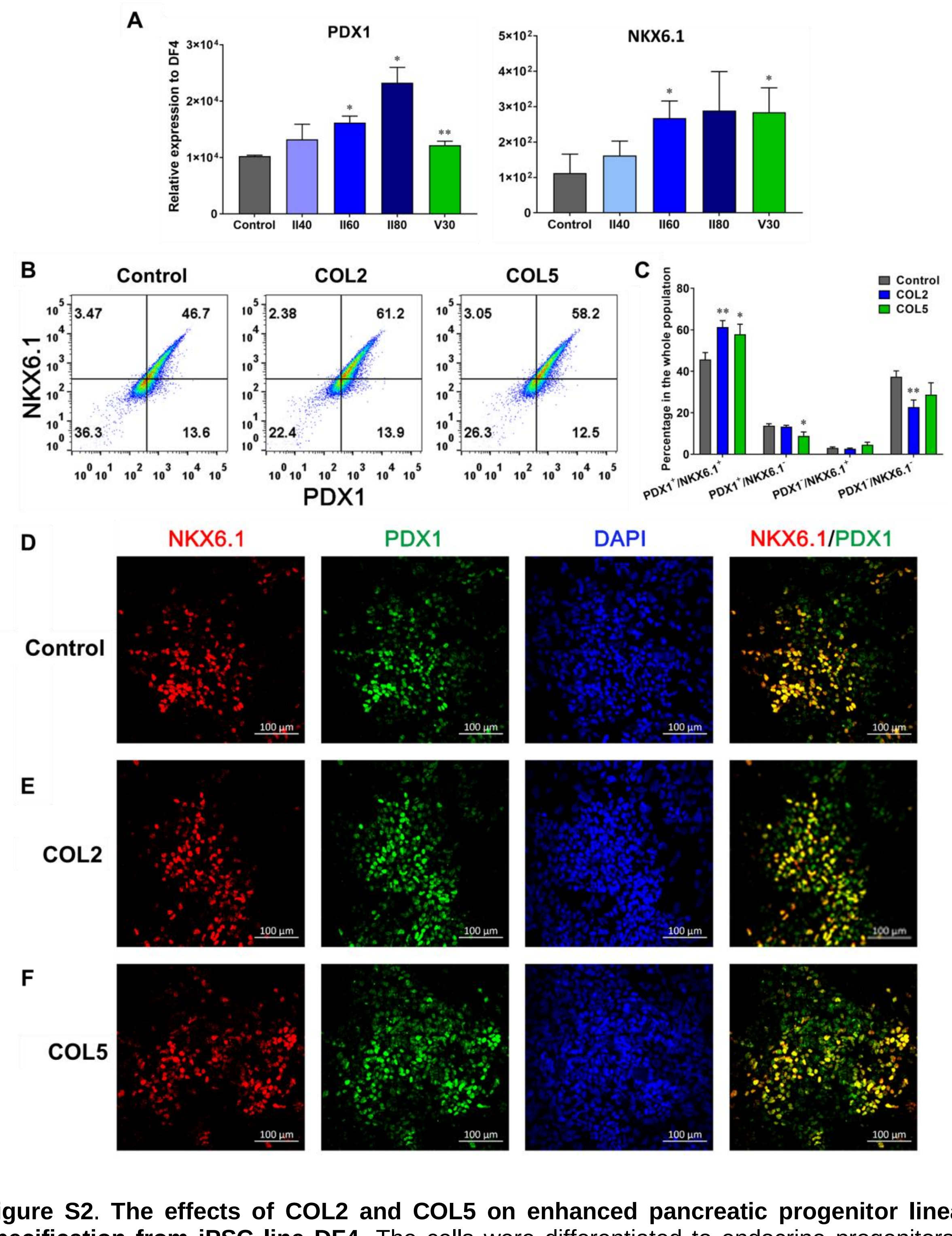

Figure S2. **The effects of COL2 and COL5 on enhanced pancreatic progenitor lineage specification from iPSC line DF4**. The cells were differentiated to endocrine progenitors on Matrigel (MG) (Control); or 30, 40, 60, or 80 μg/mL COL2 or COL5 blended Matrigel substrates. (A) The gene expression of key pancreatic progenitor markers PDX1 and NKX6.1 under COL2 or COL5 stimulation after normalizing to DF4 cells (n = 3 biological replicates in each group, except n = 4 for PDX1 of II 40 and V 30 group; n = 4 for NKX6.1 of II 40 and II 60 groups). Results were shown as mean ± SD. *, p < 0.05 and **, p < 0.01 compared to the control group. (B) Flow

cytometric analysis of PDX1⁺/NKX6.1⁺ cells formed in pancreatic progenitors. (C) The percentages of NKX6.1⁺, PDX1⁺, and PDX1⁺/NKX6.1⁺ cells in the pancreatic progenitors. Results were shown as mean ± SD (n = 3 biological replicates in each group). *, $p < 0.05$; **, $p < 0.01$. (D-F) Immunofluorescence microscopy of NKX6.1⁺, PDX1⁺, and PDX1⁺/NKX6.1⁺ cells in iPSC-derived pancreatic progenitors. Cells were counterstained with DAPI (blue). Scale bar, 100 μm.

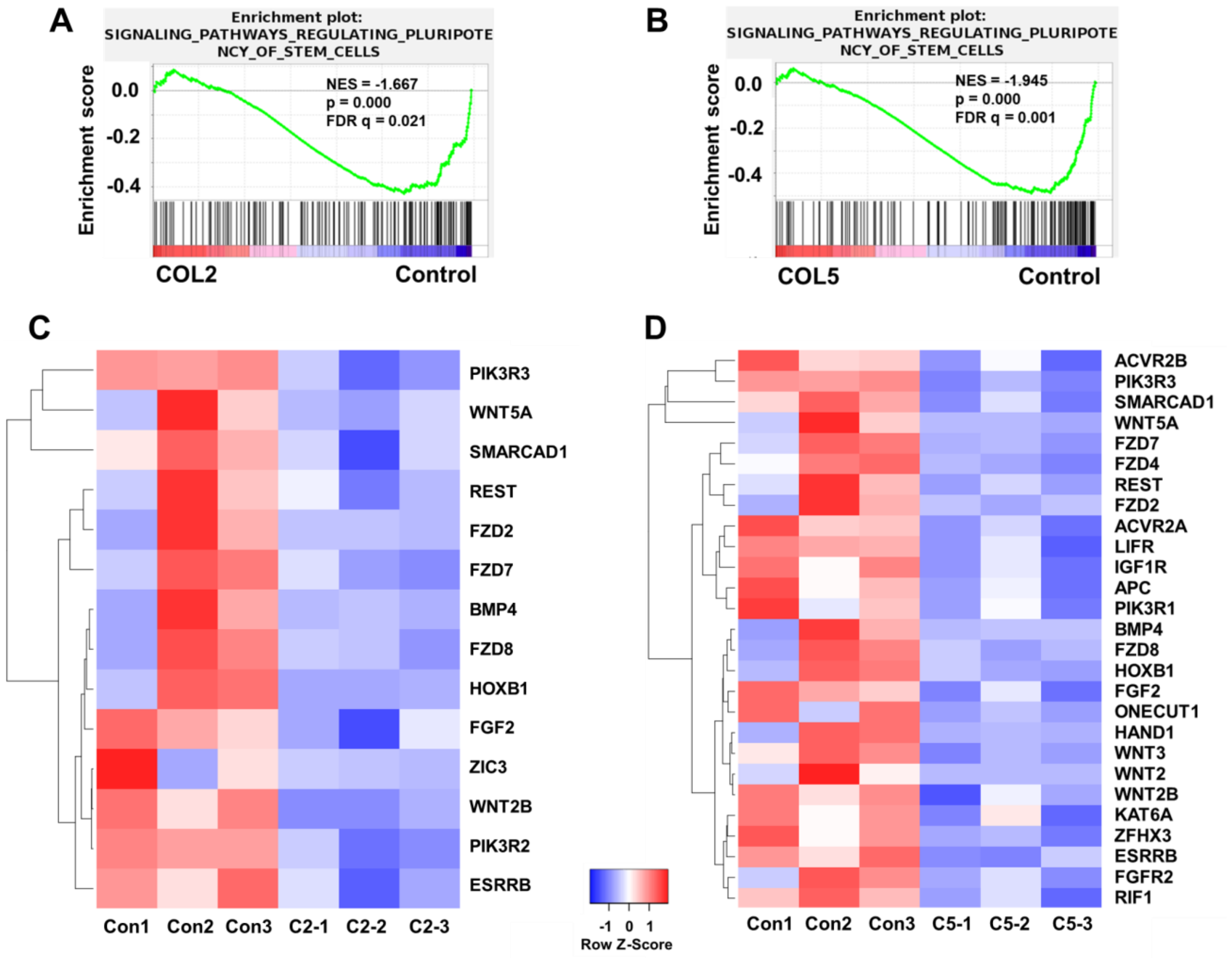

Figure S3. GSEA of downregulation of the signaling pathways that regulate the pluripotency of stem cells under COL2 (A) or COL5 (B) stimulation. (C-D) Heatmaps of significantly downregulated genes involved in signaling pathway regulating pluripotency of stem cells under the COL2 (C) or COL5 (D) stimulation ($p < 0.05$ and fold change < 0.5).

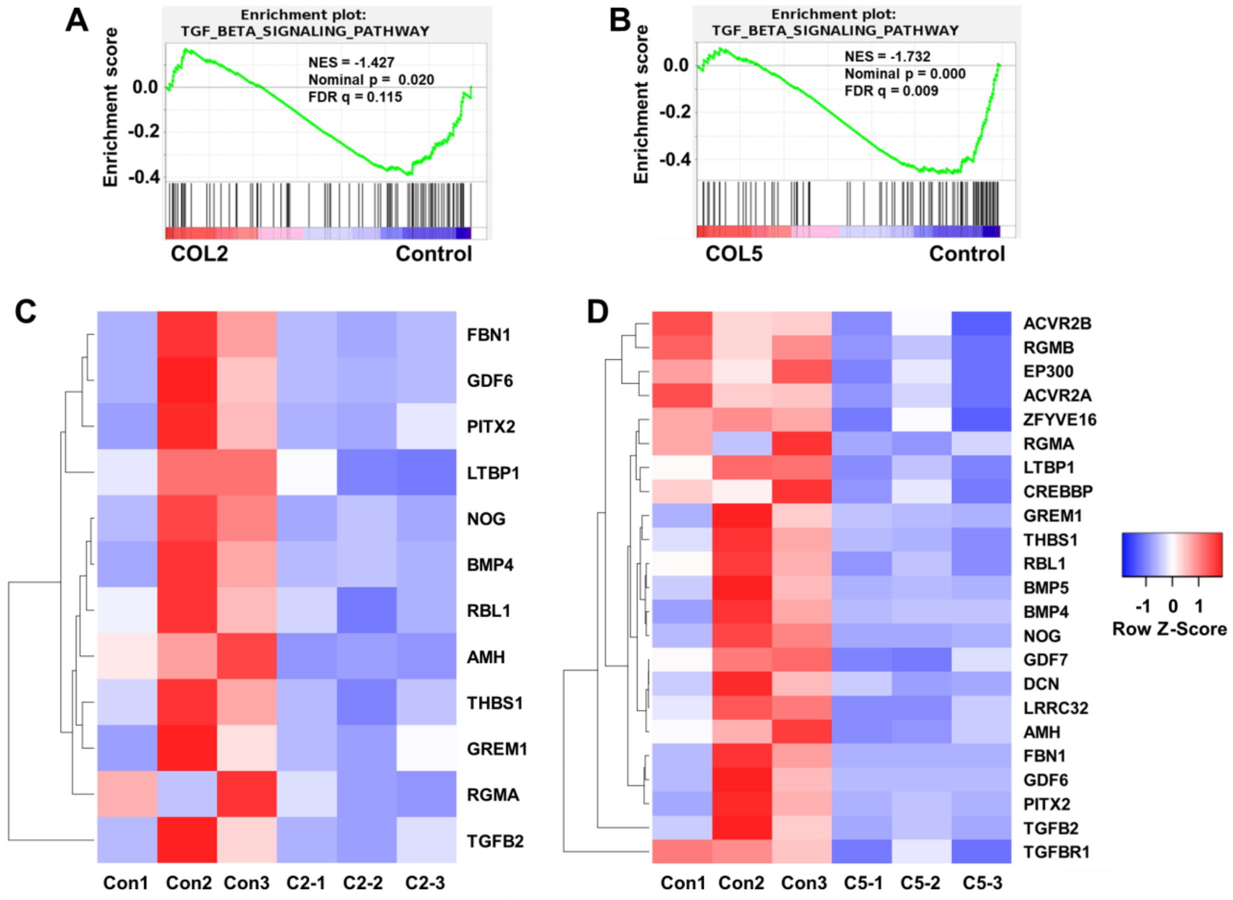

Figure S4. GSEA of downregulation of the TGF-beta signaling pathway under the COL2 (A) or COL5 (B) stimulation. (C-D) Heatmaps of significantly downregulated genes associated with the TGF-beta signaling pathway under the COL2 (C) or COL5 (D) stimulation (p < 0.05 and fold change < 0.5).

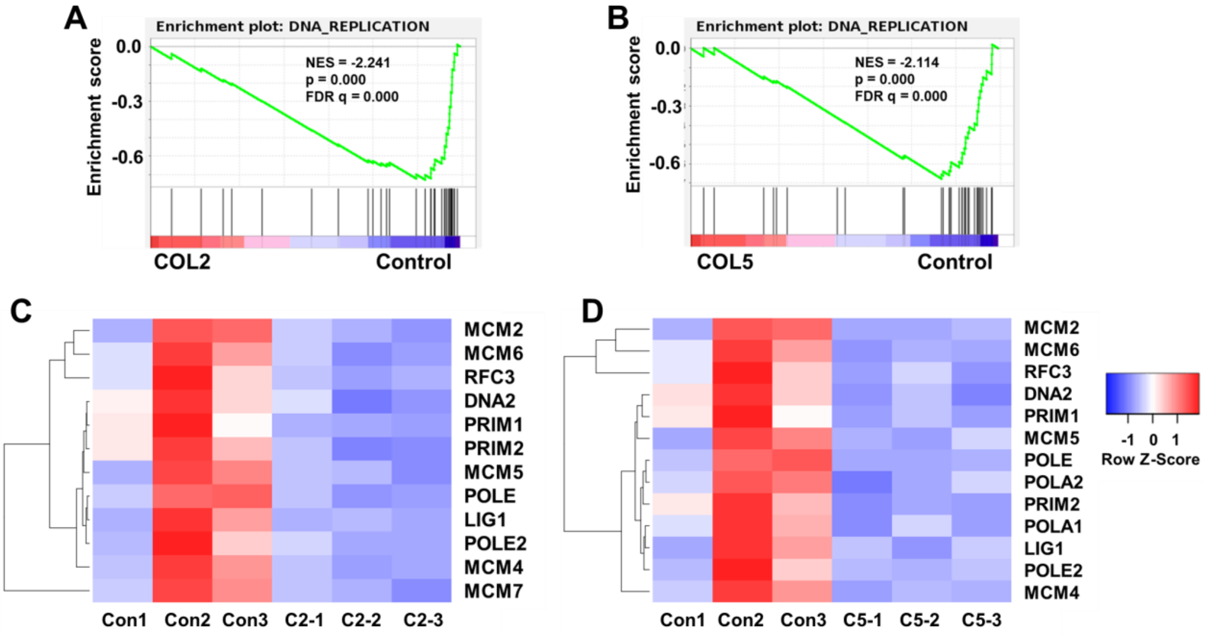

Figure S5. GSEA of downregulation of DNA replication under the COL2 (A) or COL5 (B) stimulation. (C-D) Heatmaps of significantly downregulated genes associated with the DNA replication under the COL2 (C) or COL5 (D) stimulation ($p < 0.05$ and fold change < 0.5).

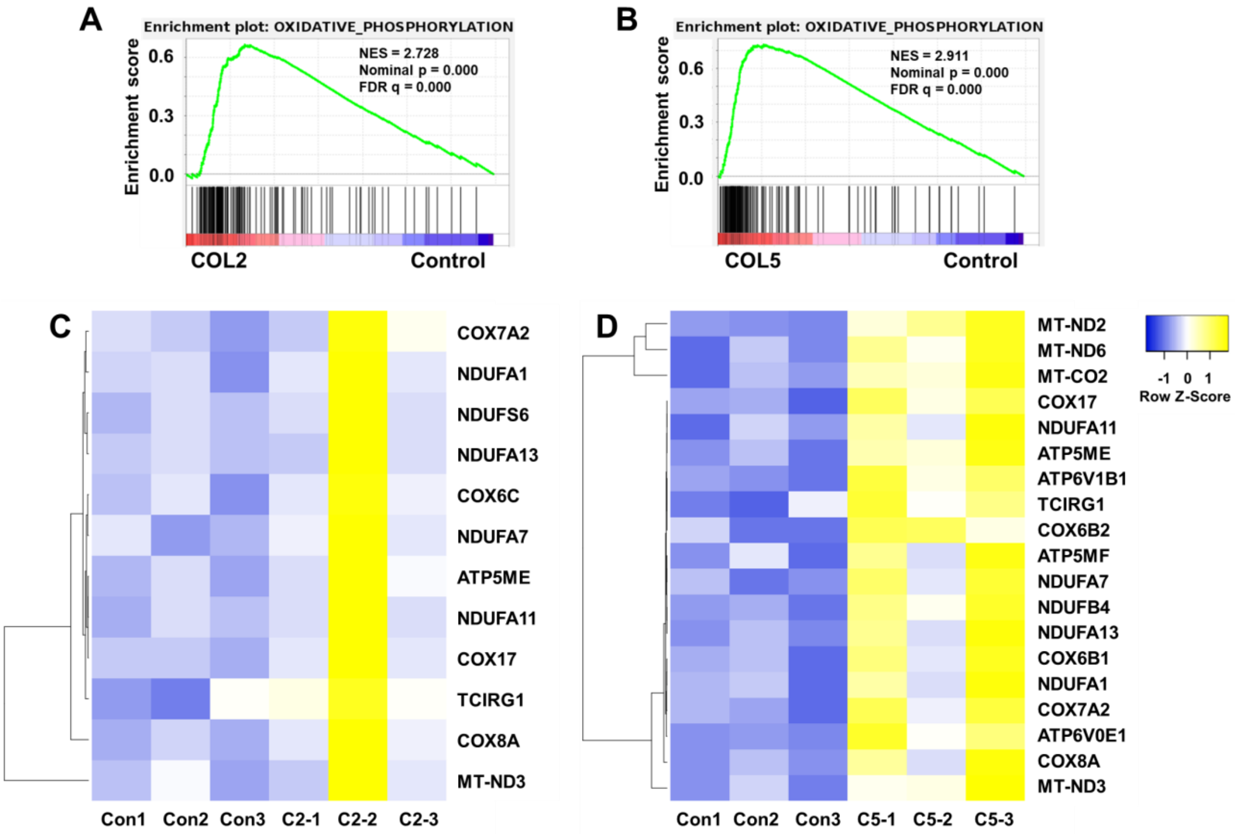

Figure S6. GSEA of upregulation of oxidative phosphorylation under COL2 (A) or COL5 (B) stimulation. (C-D) Heatmaps of significantly upregulated genes involved in the oxidative phosphorylation under the COL2 (C) or COL5 (D) stimulation ($p < 0.05$ and fold change > 2).

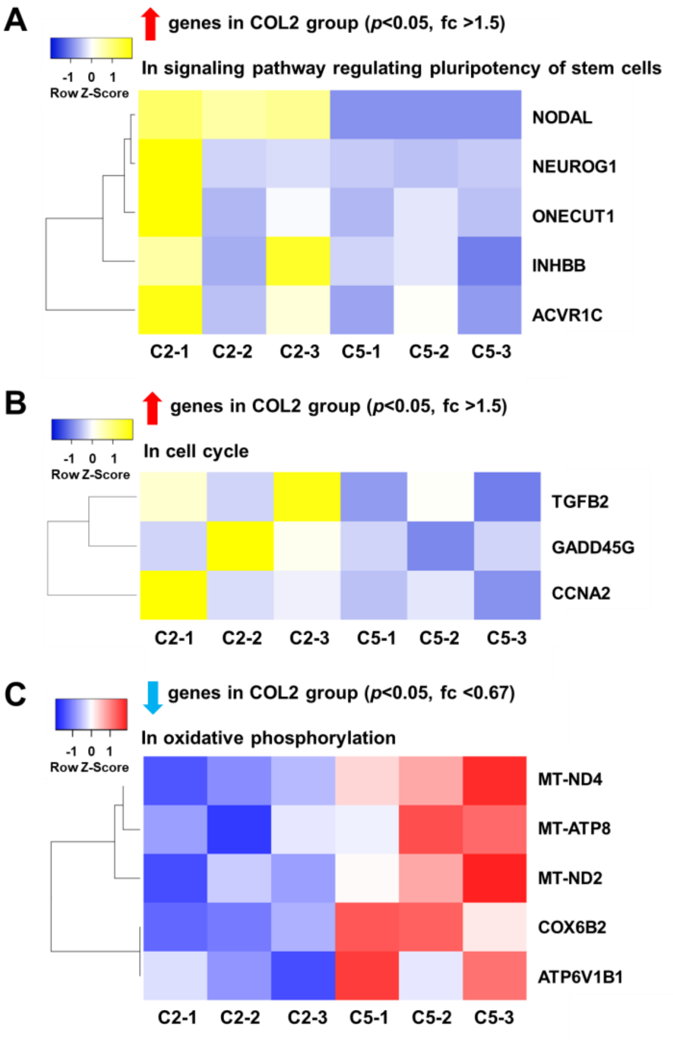

Figure S7. Comparison of the expression of gene involved in (A) signaling pathways regulating pluripotency of stem cells, (B) cell cycle, and (C) oxidative phosphorylation between COL2 and COL5 groups. They were either upregulated ($p < 0.05$, fold change >1.5) or downregulated ($p < 0.05$, fold change <0.67).

Table S1. Protocols for the regular and modified differentiation media

Stage	Duration	Protocol	Modified protocol
Stage 1: Definitive endoderm	5 days	RPMI 1640 (11 mM glucose)<br>B27<br>activin A (50 ng/mL)<br>sodium butyrate (1mM on day 1, 0.5 mM on day 2~4)	RPMI 1640 (11 mM glucose)<br>B27<br>activin A (50 ng/mL)<br>sodium butyrate (1mM on day 1, 0.5 mM on day 2~4)<br>BSA (5 g/L)

Stage 2: Posterior foregut	5 days	RPMI 1640 (11 mM glucose)<br>B27<br>ascorbic acid (0.25 mM)<br>keratinocyte growth factor (50 ng/mL)<br>retinoic acid (1 µM)<br>Noggin (100 ng/mL)<br>(−)-indolactam V (300 nM)<br>LDN193189 (100 nM)	RPMI 1640 (11 mM glucose)<br>B27<br>ascorbic acid (0.25 mM)<br>keratinocyte growth factor (50 ng/mL)<br>retinoic acid (1 µM)<br>Noggin (100 ng/mL)<br>(−)-indolactam V (300 nM)<br>LDN193189 (100 nM)<br>BSA (5 g/L)
Stage 3: Endocrine progenitor	5 days	DMEM/F12 (17.5 mM glucose)<br>B27<br>ascorbic acid (1 µM)<br>(−)-indolactam V (300 nM)<br>LDN193189 (200 nM)<br>3,3’,5-Triiodo- L-thyronine sodium salt (T3) (1 µM)<br>RepSox (10 µM)<br>heparin (10 µg/mL)<br>glucose (final 20 mM)	DMEM/F12 (17.5 mM glucose)<br>B27<br>ascorbic acid (1 µM)<br>(−)-indolactam V (300 nM)<br>LDN193189 (200 nM)<br>T3 (1 µM)<br>RepSox (10 µM)<br>heparin (10 µg/mL)<br>glucose (final 20 mM)<br>BSA (5 g/L)<br>ITX-S (1%)<br>sodium pyruvate (0.5 mM)
Stage 4: Endocrine lineage	7 days	RPMI 1640 (11 mM glucose)<br>B27<br>T3 (1 µM)<br>RepSox (10 µM)<br>heparin (10 µg/mL)<br>N-acetyl cysteine (1 mM)<br>R428 (0.5 µM)<br>trolox (10 µM)<br>γ-secretase inhibitor XX (100 nM)<br>nicotinamide (10 mM)<br>angiopoietin-2 (20 ng/mL)<br>glucose (final 20 mM)	RPMI 1640 (11 mM glucose)<br>B27<br>T3 (1 µM)<br>RepSox (10 µM)<br>heparin (10 µg/mL)<br>N-acetyl cysteine (1 mM)<br>R428 (0.5 µM)<br>trolox (10 µM)<br>γ-secretase inhibitor XX (100 nM)<br>nicotinamide (10 mM)<br>angiopoietin-2 (20 ng/mL)<br>glucose (final 20 mM)<br>$ZnSO_4$ (10 µM)<br>BSA (5 g/L)<br>ITX-S (1%)<br>sodium pyruvate (0.5 mM)
Stage 5: Mature endocrine cells	7-10 days	CMRL1066 supplemented<br>T3 (1 µM)<br>RepSox (10 µM)<br>γ-secretase inhibitor XX (100 nM)<br>nicotinamide (10 mM)<br>angiopoietin-2 (20 ng/mL)	CMRL1066 supplemented<br>T3 (1 µM)<br>Rep (10 µM)<br>γ-secretase inhibitor XX (100 nM)<br>nicotinamide (10 mM)<br>angiopoietin-2 (20 ng/mL)<br>BSA (5 g/L)<br>ITX-S (1%)<br>FBS (10%)<br>sodium pyruvate (0.5 mM)

Table S2. Primers and probes for TaqMan qRT-PCR

Genes	Sequences of primers and probes (5' to 3') or Assay IDs from Applied Biosystems
FOXA2	Forward: 5'-CCGACTGGAGCAGCTACTATG -3'<br>Reverse: 5'-TACGTGTTCATGCCGTTCAT -3'<br>Probe: 5'-FAM-CAGAGCCCGAGGGCTACTCCTCC-BHQ-3'
HES1	Hs00172878_m1
HNF4A	Hs00230853_m1
ISL1	Hs00158126_m1
NKX6.1	Hs00232355_m1
NGN3	Forward: 5'-TCTCTATTCTTTTGCGCCGG-3'<br>Reverse: 5'-CTTGGACAGTGGGCGCAC-3'<br>Probe: 5'-FAM-AGAAAGCTCG-BHQ-3'
PDX1	Forward: 5'-CCTTTCCCATGGATGAAGTC-3'<br>Reverse: 5'-CGTCCGCTTGTTCTCCTC-3'<br>Probe: 5'-FAM-AAGCTCACGCGTGGAAAGGCC-BHQ-3'
PTF1A	Forward: 5'-CAGGCCCAGAAGGTCATC-3'<br>Reverse: 5'-GGGAGGGAGGCCATAATC-3'<br>Probe: 5'-FAM-ATCTGCACCC-BHQ-3'
SOX9	Hs00165814_m1
PPIA	Hs99999904_m1

Table S3. Antibodies used in flow cytometry

Antibodies	Host	Manufacturer	Catalogue #	Dilution
SOX17	Goat	R&D SYSTEMS	IC1924A	1:10
Goat IgG	Goat	R&D SYSTEMS	IC108A	1:10
NKX6.1	Mouse	DSHB at U. of Iowa	F55A12-s	1:30
Mouse IgG	Goat	Sigma	SAB4600397	1:600
PDX1	Mouse	BD Biosciences	562161	1:20
Mouse IgG	Mouse	BD Biosciences	555749	1:80

Table S4. Antibodies used in immunostaining microscopy

Antibodies	Host	Manufacturer	Catalogue #	Dilution
NKX6.1	Mouse	DSHB at U. of Iowa	F55A12-s	1:50
Mouse IgG	Goat	Sigma	SAB4600397	1:500
PDX1	Mouse	BD Biosciences	562161	1:50
Mouse IgG	Mouse	BD Biosciences	555749	1:200
C-peptide	Rat	University of Iowa	GN-ID4-s	1:30
Rat IgG	Donkey	Sigma	SAB4600156	1:300
Glucagon	Rabbit	Sigma	SAB1306596	1:50
Rabbit IgG	Goat	Sigma	F9887	1:500
MAFA	Rabbit	Abcam	ab26405	1:250
MAFB	Rabbit	Millipore	ABE55	1:100
Glucagon	Mouse	R&D SYSTEMS	MAB1249	1:50
Somatostatin	Rat	Millipore	MAB354	1:100
Rat IgG	Goat	Invitrogen	A-11006	1:300
Pancreatic Polypeptide	Mouse	R&D SYSTEMS	MAB62971	1:50
Mouse IgG	Donkey	Sigma	SAB4600035	1:200

Table S5. Antibodies used in immunoblotting

Antibodies	Host	Manufacturer	Catalogue #	Dilution
β-catenin	Rabbit	Sigma	PLA0230	1:2000
MYC	Rabbit	Novus	NB600-336SS	1:1000
DDIT4	Rabbit	Novus	NBP1-77321SS	1:1000
ICAM1	Mouse	Novus	NBP2-22541	1:1000
PD-L1	Rabbit	Novus	NBP1-76769	1:1000
Rabbit IgG-HRP	Goat	Cell Signaling	7074P2	1:15000
Mouse IgG-HRP	Goat	ThermoFisher	PI31431	1:5000

β-actin	Mouse	Sigma	A3854	1:30000
